# Genetic Engineering of *Crypthecodinium cohnii* to Increase Growth and Lipid Accumulation

**DOI:** 10.3389/fmicb.2018.00492

**Published:** 2018-03-19

**Authors:** Jinjin Diao, Xinyu Song, Xiaoqing Zhang, Lei Chen, Weiwen Zhang

**Affiliations:** ^1^Laboratory of Synthetic Microbiology, School of Chemical Engineering and Technology, Tianjin University, Tianjin, China; ^2^Key Laboratory of Systems Bioengineering (Ministry of Education), Tianjin University, Tianjin, China; ^3^SynBio Research Platform, Collaborative Innovation Center of Chemical Science and Engineering, Tianjin University, Tianjin, China; ^4^School of Environmental Science and Engineering, Tianjin University, Tianjin, China; ^5^Center for Bio-safety Research and Strategy, Tianjin University, Tianjin, China

**Keywords:** genetic modification, DHA, RuBisCO, *C. cohnii*, metabolomic analysis

## Abstract

In this study, we evaluated suitable selected markers and optimized transformation protocols to develop a new genetic transformation methodology for DHA-producing *Crypthecodinium cohnii*. Additionally, ribulose 1,5-bisphosphate carboxylase/oxygenase (RuBisCO), potentially involved in CO_2_ fixation under autotrophic conditions, was selected as the target for construction of a gene knockdown mutant. Our results show that the constructs were successfully inserted into the *C. cohnii* chromosome by homologous recombination. Comparative analysis showed that deletion of the RuBisCO gene promoted cell growth and increased the lipid content of *C. cohnii* under heterotrophic conditions compared with those of the wild-type. The liquid chromatography-mass spectrometry (LC-MS) based metabolomic analysis showed that the metabolites involved in energy metabolism were upregulated, suggesting that the deletion of the RuBisCO gene may contribute to the re-direction of more carbon or energy toward growth and lipid accumulation under heterotrophic conditions.

## Introduction

The interest in the omega-3 family of long-chain polyunsaturated fatty acids (PUFAs) has increased greatly because these compounds exert recognized beneficial effects on human health (Hornstra, 2000; De Swaaf et al., 2003). Among them, docosahexaenoic acid (DHA) is an important structure component of neural and retinal tissues (Agostoni et al., 1995; Weete et al., 1997). DHA is also a key fatty acid component in breast milk, and is necessary for brain development in infants (Sijtsma and de Swaaf, 2004; Kuratko and Salem, 2013). DHA is widely used in various infant food products (Barclay et al., 1994). The inherent defects of traditional sources of DHA, which is extracted from deep-sea fish oil, limits its application in infant foods (Carlson, 1996). It is therefore necessary to develop alternative sources and technologies for DHA production.

The heterotrophic non-photosynthetic dinoflagellate microalga *Crypthecodinium cohnii* accumulates lipids with a high fraction of DHA, and is widely used in industrial fermentation for algal oil and DHA production (Harrington and Holz, 1968; Bell and Henderson, 1990; Jiang et al., 1999; de Swaaf et al., 2003; Ratledge, 2004). Under optimized cultivation conditions, *C. cohnii* accumulates less than 1% of the other type of PUFAs (de Swaaf et al., 1999; De Swaaf et al., 2003); this shows remarkable advantages for the downstream DHA purification process. Recently, much effort has been placed into improving DHA accumulation in *C. cohnii*. For example, optimization of the fermentation parameters in fed-bath experiments led to a production of 109 g/L dry biomass, 61 g/L lipid, and a 19 g/L DHA in *C. cohnii* (de Swaaf et al., 1999, 2003; De Swaaf et al., 2003; Sijtsma and de Swaaf, 2004). In addition, chemical triggers such as butylated hydroxyanisol (BHA) (Sui et al., 2014) and ethanolamine (Li et al., 2015) were applied to directly stimulate lipid accumulation in *C. cohnii*. However, no study thus far has reported on using genetic engineering on DHA-producing *C. cohnii*. Several studies have successfully applied genetic engineering to improve lipid content in various microalgae (Radakovits et al., 2011; Hamilton et al., 2014; Iwai et al., 2014; Avidan et al., 2015). Thus, it is necessary to develop relevant methodologies for genetic engineering of *C. cohnii* with high-efficiency production of DHA. Recently, transformation systems have been developed for several other dinoflagellate species, such as *Amphidinium* and *Symbiodinium* (Te and Miller, 1998); however, no transformation system has yet been established for *C. cohnii*.

High-efficiency production of target products requires re-balancing of energy and carbon flux in cells (Komatsu et al., 2010). For example, lipid production is maximized in *Yarrowia lipolytica* by engineering a cytosolic redox metabolism to increase the supply of NADPH (Qiao et al., 2017). Limonene production is enhanced by portioning a greater carbon flux to 2-*C*-methyl-D-erythritol 4-phosphate pathway (Wang et al., 2016). *C. cohnii* can be derived from a photosynthetic ancestor and harbors a reduced plastid (Sanchez-Puerta et al., 2007). The ribulose-1,5-bisphosphate carboxylase/oxygenase (RuBisCO) in non-photosynthetic *C. cohnii*, cultivated under heterotrophic conditions, is still transcribed (Sanchez-Puerta et al., 2007). Typically, RuBisCO is an enzyme involved in the first major step of carbon fixation, a process by which atmospheric carbon dioxide is converted to energy-rich molecules such as glucose (Dhingra et al., 2004). In addition, RuBisCO may act as an oxygenase in photorespiration, and is involved in glycine and serine biosynthesis (Leegood, 2007). The energy source ATP and reducing equivalent NADPH are consumed in the two aforementioned reactions. To date, the specific roles of RuBisCO in non-photosynthetic *C. cohnii* are not clearly elucidated. However, with respect to the metabolic economy of a cell, pathways involved in CO_2_ fixation and photorespiration may not be essential under heterotrophic conditions. For example, *Rhodobacter sphaeroides* and *Rhodopseudomonas palustris*, the RuBisCO gene deletion mutants, can save more energy and reductants under chemoheterotrophic conditions (Laguna et al., 2011). Therefore, we hypothesized that in *C. cohnii*, the deletion of these high abundance but non-essential genes, such as RuBisCO, may optimize the carbon flux and redirect the energy flux (ATP and NADPH) that are consumed in the Calvin-Benson-Bassham (CBB) cycle of cell growth and lipid accumulation under heterotrophic conditions.

To engineer *C. cohnii* for high-efficiency lipid and DHA production, we first developed a genetic transformation system for *C. cohnii*, and then applied for the construction of a RuBisCO-deleted strain. Our results show that deletion of the RuBisCO gene promoted cell growth and increased lipid content of *C. cohnii* under heterotrophic growth conditions compared with those of the wild-type. To explore the possible mechanism, a liquid chromatography-mass spectrometry (LC-MS) based metabolomics analysis was used to compare a RuBisCO-deleted and wild-type *C. cohnii*; this analysis provided new insights into the metabolic changes associated with increased growth and lipid accumulation in *C. cohnii*. The obtained information will be useful for engineering high-efficiency lipid and DHA-producing strains in the future.

## Materials and Methods

### Algae Strains and Cultivation

*Crypthecodinium cohnii* ATCC 30556 was obtained from American Type Culture Collection (ATCC) and cultivated in a basal medium according to Sui et al. (2014). The seed cultures were cultivated in 50 mL of basal medium (pH 6.5) containing 9.0 g/L glucose, 2.0 g/L yeast extract (OXOID, Basingstoke, United Kingdom), and 25.0 g/L sea salt (Sigma-Aldrich, St. Louis, MO, United States), in 250-mL Erlenmeyer flasks at 25°C, shaken at 180 rpm.

### Preparation of *C. cohnii* Competent Cells

*Crypthecodinium cohnii* cells (10 mL) in exponential phase were harvested by centrifugation at 3500 rpm (Eppendorf 5430R, Hamburg, Germany) at 4°C for 5 min. The collected cells were re-suspended in 1 mL of 25 mM DTT and pretreated at 30°C for 15 min. After the pretreatment, cells were harvested again by centrifugation at 3500 rpm (Eppendorf 5430R, Hamburg, Germany) at 4°C for 5 min, and washed three times with 10 mL of 1 M sorbitol. Finally, cells were re-suspended in 1 mL of 1 M sorbitol.

### Electroporation With FITC-Dextran as Fluorescence Marker

The final mixture of competent cells (200 μL), 1 μL sperm DNA (100 mg/mL), and 1 μg FITC-Dextran (70 kDa) were transferred into a pre-chilled 2 mm gap electroporation cuvette (Bio-Rad, Hercules, CA, United States), ice bathed for 5 min, after which different electric fields were applied *via* electroporation system (Gene Pulser Xcell; Bio-Rad, Hercules, CA, United States). After the electric shock was applied, the cells in the cuvette were washed twice with basal medium, re-suspended in 2 mL basal medium, and treated with 1 mg/mL trypsin at 37°C for 15 min (Richard et al., 2003).

### Analysis of Viability and Fluorescence

Fluorescence intensity in the transformed cells was measured by a spectrophotometer (F-2700EL, HITACHI, Chiyoda, Japan). The excitation wavelength for FITC-Dextran was 490 nm; the highest peak at the emission wavelength for FITC-Dextran (520 nm) was used to calculate the transformation efficiency. The transformed cells were also observed under a fluorescence microscope (OLYMPUS, BX43, Shinjuku, Japan).

### Antibiotics Sensitivity Assessment of *C. cohnii*

*Crypthecodinium cohnii* cells (50 μL) of the exponential phase were selected on plates containing different concentrations of hygromycin (10–50 mg/L) or bleomycin (10–200 mg/L), and cultivated at 25°C for up to 2 weeks.

### Constructs Used for Transformation

*Phusion* high-fidelity DNA polymerase (Thermo-Fisher, Waltham, MA, United States) was used in the PCR reactions. A fusion PCR-based method was employed for the construction of gene knockout fragments (Wang et al., 2002). For the target genes selected, three sets of primers were designed to amplify a linear DNA fragment containing the hygromycin or bleomycin resistance gene with two flanking arms of DNA upstream and downstream of the targeted gene. The linear fused PCR amplicon was used directly for transformation into *C. cohnii* by electroporation. Hygromycin and bleomycin resistance genes were amplified from pCAMBIA1301 (Hajdukiewicz et al., 1994) and pSP124s (Lumbreras et al., 1998), respectively. PCR primers used for mutant construction are listed in **Supplementary Table S1**.

### Transformation of *C. cohnii*

A 200-μL mixture of competent cells (prepared as mentioned in section “Electroporation With FITC-Dextran as Fluorescence Marker”), 1 μL sperm DNA (100 mg/mL), and 1∼2 μg DNA fragment were transferred into a 2-mm pre-chilled electroporation cuvette and ice bathed for 5 min. Gene Pulser Xcell (Bio-Rad, Hercules, CA, United States) was used for electroporation, and the electroporator was adjusted to 2000 V field strength and 50 μF capacitance. After electroporation, the cells were recovered in 2 mL of basal medium, and cultivated for 48 h at 25°C while shaking at 180 rpm. Then, 50 μL of cell suspension were selected on basal medium plates containing antibiotics of suitable concentration, as described in section “Antibiotics Sensitivity Assessment of *C. cohnii.*”

### Mutant Screening and Analysis

Hygromycin or bleomycin-resistant transformants on the plates were streaked onto fresh basal medium plates supplemented with hygromycin or bleomycin, and passaged several times. Mutants were confirmed by PCR and sequencing analysis. TIANamp Bacterial DNA Kit (TianGen, Beijing, China) was used to extract chromosomal DNA of wild-type and mutant *C. cohnii*. PCR primers used for validating mutants are listed in **Supplementary Table S1**.

### Comparative Growth Analysis and LC-MS Targeted Metabolomics

Cell density of wild-type and mutant *C. cohnii* was determined by a UV-1750 spectrophotometer (Shimadzu, Kyoto, Japan) at OD_490_. For LC-MS metabolomic analysis, all reagents, including standard metabolites, were obtained from Sigma-Aldrich (Sigma-Aldrich, St. Louis, MO, United States). Cells were harvested by centrifugation at 8000 × *g* for 8 min at 25°C (Eppendorf 5430R, Hamburg, Germany). Metabolite extraction and LC-MS analysis were performed according to a previously published protocol (Li et al., 2015). Metabolomic data were normalized by interior control and cell number, and then submitted to principal component analysis (PCA) using SIMCA-P 11.5.

### Semi-quantitative and Quantitative RT-PCR Analyses

(i) Semi-quantitative RT-PCR analysis: total RNA was extracted from cells with Trizol reagent (Invitrogen, Camarillo, CA, United States) according to the manufacturer’s instructions. RT-PCR was conducted using cDNAs generated from 1 μg of total RNA with a RevertAid First Strand cDNA Synthesis Kit (Thermo-Fisher, Waltham, MA, United States). The RT-PCR exponential phase was determined using 22–30 cycles, to allow for semi-quantitative comparisons of cDNA developed using identical reactions with Taq polymerase (Tiangen, Beijing, China); the method is described in Song et al. (2016). The housekeeping gene 18S rDNA (GenBank accession no. FJ821501.1) of *C. cohnii* was used as internal reference.

(ii) Quantitative RT-PCR analysis was conducted according to the method described previously (Song et al., 2016). Briefly, a StepOne Plus Real-Time PCR (Applied Biosystems, Foster City, CA, United States) was used to conduct the analysis. Cell pellets were re-suspended in Trizol reagent (Invitrogen, Camarillo, CA, United States) and mixed thoroughly by vortexing. Total RNA extraction was achieved using a miRNeasy Mini Kit (Qiagen, Valencia, CA, United States). Contaminating DNA in RNA samples was removed with DNase I according to instructions in the miRNeasy Mini Manual (Qiagen, Valencia, CA, United States). The RNA quality and quantity were determined using NanoDrop 2000 (Thermo-Fisher, Waltham, MA, United States) and subjected to cDNA synthesis. Then, 2^-ΔΔ*C*_T_^ was used to estimate the relative abundance of different mRNA molecules: the higher the ^Δ^C_T_ value, the less abundant is the corresponding mRNA (Livak and Schmittgen, 2001).

### Enzyme Activity Assay and Western Blotting

RuBisCO enzyme activity was determined using the ^14^C isotope-based method described by Lorimer et al. (1977). Afterward, protein extraction was conducted according to Lilley et al. (2010). The assay was performed in a 460-μL final volume containing 50 mM Tris (adjusted to pH 8.1 with HCl at 20°C), 10 mM EDTA, 20 mM MgCl_2_, 10 mM NaH^14^CO_3_ (Sigma-Aldrich, St. Louis, MO, United States), and 20 μL crude extracts. The reaction was started by adding 20 μL of 10 mM RuBP (Sigma-Aldrich, St. Louis, MO, United States). The reaction was stopped after 1 min by adding 200 μL of 2 M HCl. The contents of the vial were quantitatively transferred to a scintillation vial, dried, and quantified per a previously described protocol (Sudhani et al., 2015).

For western blotting assay, total protein extraction was conducted according to Lilley et al. (2010). Then, 10 μl of loading buffer (Tiangen, Beijing, China) was added to 20 μl of crude extracts, and the mixture was heated for 10 min at 100°C. The samples were loaded onto a precast polyacrylamide gel. After SDS-PAGE, the separated proteins were electroblotted onto a polyvinylidene fluoride (PVDF) membrane and probed with an affinity-purified antibody against the large subunit of RuBisCO (AS03 037, Agrisera, Vännäs, Sweden). After incubating with a secondary antibody [horseradish peroxidase-conjugated goat anti-rabbit IgG; AS 09602, Agrisera, Vännäs, Sweden], ECL Prime (GE, Amersham, United Kingdom) was used for detection. The detection analysis was conducted using a CCD camera according to the manufacture’s instructions.

### Lipid Extraction and Analysis

*Crypthecodinium cohnii* cells were cultivated in basal medium with 21 g/L glucose, harvested at 84 h by centrifugation (3500 × *g*) (Eppendorf 5430R, Hamburg, Germany), and freeze-dried to generate a lyophilized algal powder. Lipid extraction and analysis were conducted based on the method described by Li et al. (2015).

### Statistical Analysis

All experiments were conducted with at least three biological replicates to ensure reproducibility. Statistical analyses were performed using a one-way analysis of variance (ANOVA) with subsequent *post hoc* multiple-comparison LSD tests, which were calculated using SPSS Statistics software. Statistically significant values were defined as *P* < 0.05.

## Results and Discussion

### Establishing a Transformation Protocol

**FIGURE 1:**
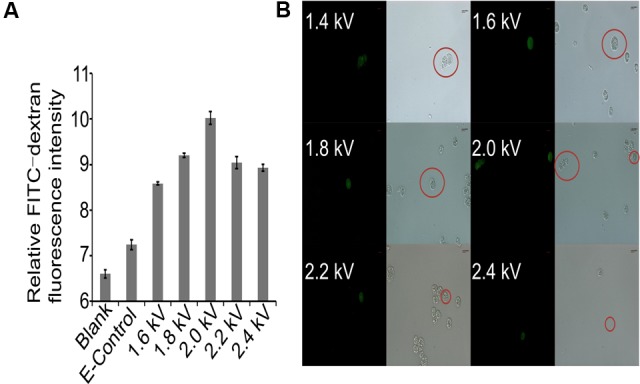
Evaluation of transformation protocol. **(A)** Measurement of relative FITC-dextran fluorescence intensity in *C. cohnii* using different electric fields. Blank, FITC-dextran untreated cells; E-Control, FITC-dextran treated cells without electric shock. Three independent biological replicates were used, and statistical significance was defined as *p*-value less than 0.05; **(B)** Microscopic examination of transformed *C. cohnii* cells. Scale bar = 8.49 μm.

*Crypthecodinium cohnii*, a heterotrophic dinoflagellate alga, is an important microalgal species for industrial fermentation of algal oil and DHA production. However, because of the lack of genetic transformation systems, no genetic engineering work has been conducted on this valuable species. This has restricted the efforts in strain improvement and deciphering the mechanism of DHA accumulation in *C. cohnii*. Macromolecules are successfully transferred into protoplasts or spheroplasts of *C. cohnii* by liposomal-mediated transformation (Kwok et al., 2007). However, preparation of protoplasts or spheroplasts can be challenging and time consuming. Electroporation is a powerful method for genetic engineering of microalgae (Sun et al., 2005; Weeks, 2011). To establish an efficient transformation system for *C. cohnii*, we first selected the macromolecule FITC-Dextran (70 kDa) as a fluorescent marker to evaluate the electroporation parameters for *C. cohnii*. As shown in **Figure 1A**, the fluorescence values of the transformed cells (1.6 kV, 200 Ω, 50 μF) were slightly increased when compared with those of the control cells, suggesting that the macromolecule FITC-Dextran was delivered into *C. cohnii* cells by electroporation. The results of examination via fluorescence microscopy (**Figure 1B**) confirmed that the increase of fluorescence values was due to the delivery of FITC-Dextran into *C. cohnii* cells. To improve transformation efficiency, we further optimized the electroporation parameters by applying voltages ranging from 1.6 to 2.4 kV, because higher electric shocks can lead to more uptake of extracellular molecules by the cells (Jeon et al., 2013). When voltage was adjusted to 2.0 kV, we observed the maximum intensity of fluorescence; however, further increase of voltage to 2.2 or 2.4 kV led to a decrease of fluorescence intensity in the cells (**Figure 1A**). According to our optimization analysis using FITC-Dextran, an electroporation protocol with parameters of 2.0 kV, 200 Ω, and 50 μF was determined for the next procedure using *C. cohnii* cells.

### Transformation of *C. cohnii*

**FIGURE 2:**
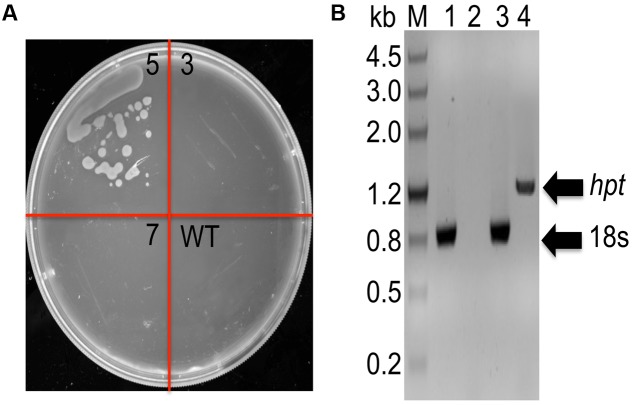
Analysis of hygromycin-resistant *C. cohnii* mutants. **(A)** Phenotypic analysis of transformed and WT cells on a plate containing 40 mg/L hygromycin. WT, wild-type *C. cohnii* cells; 3, 5, and 7 are clones streaked on selective plate, and the clone 5 is the positive clone demonstrated below in **(B)**; **(B)** Genomic PCR analysis of the mutants, conducted by detecting the hygromycin resistance gene. M, DNA marker III; lane 1 and 2: WT genome was used as genomic PCR template; lane 3 and 4: the genome of strain 5 was used as the genomic PCR template. The primers used for the 18s gene were 18s-F1 and 18s-R; the primers used for the *hyg* gene were P-F and Hyg-R2.

In order to determine whether DNA constructs could also be transferred and integrated by homologous recombination (HR) into *C. cohnii* chromosomal DNA, we selected gene encoding 18S rDNA as a suitable site for HR (Cheng et al., 2010; Yan et al., 2013). 18S rDNA exists in multiple copies in the chromosomes and is located at transcriptionally active regions in organisms. This renders 18S rDNA more suitable for integration of foreign DNA than other sites. In addition, insertion inactivation of at least some copies of 18S rDNA is not lethal (Pisabarro et al., 1998). However, it is necessary to identify appropriate selectable makers for transformation. As shown in **Supplementary Figure S1**, when the concentrations of hygromycin and bleomycin were increased to 40 mg/L and 200 mg/L, respectively, no growth was observed on the plates containing the antibiotics; this suggests that 40 mg/L hygromycin and 200 mg/L bleomycin can be suitable concentrations used for selection, respectively. The transformation construct C1 carried the *hygR* resistant gene, which conferred resistance to hygromycin as a selection marker (**Supplementary Figure S2A**). For transformation, 2 μg of construct C1 DNA was used, which gave three possible positive clones (i.e., strain 3, 5, 7). After streaking the clones onto fresh basal medium in a plate containing 40 mg/L hygromycin, growth was observed only for strain 5 (**Figure 2A**). To further validate whether the selected marker gene *hygR* was successfully integrated into the genome, we conducted PCR with primer sets (P-F and Hyg-R2) to detect the *hygR* selected marker gene using chromosomal DNA as template (**Figure 2B**). The amplicon was purified, sequenced, and confirmed. The results demonstrated the integration of *hygR* gene into the *C. cohnii* genome (data not shown), suggesting a successful establishment of the *C. cohnii* transformation protocol. The detailed transformation protocol is provided in **Supplementary Figure S3**.

### Construction of RuBisCO-Deletion Mutants

**FIGURE 3:**
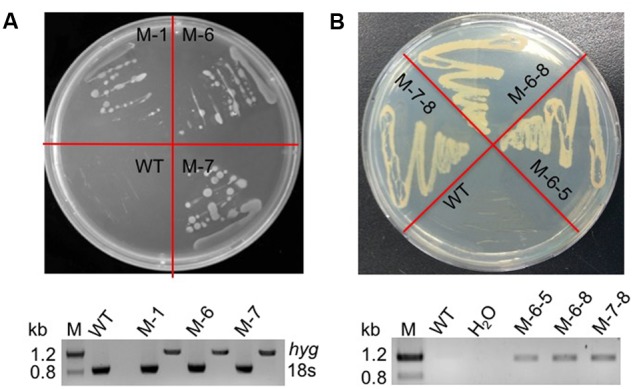
Verification of the mutants by phenotype and PCR. **(A)** Phenotypic and PCR analysis of the RuBisCO single-deletion mutant. Top panel: WT and transformed cells on a plate containing 40 mg/L hygromycin; bottom panel: PCR detection of the hygromycin resistance gene in the mutant; the primers used were P-F and Hyg-R2. **(B)** Phenotypic and PCR analysis of the RuBisCO double-deletion mutant. Top panel: WT and transformed cells on a plate containing 40 mg/L hygromycin and 200 mg/L bleomycin; bottom panel: PCR detection of the bleomycin resistance gene in the mutants; the primers used were Ble-F3 and Ble-R2. H_2_O was used as negative control to exclude contamination of the PCR reagents.

**FIGURE 4:**
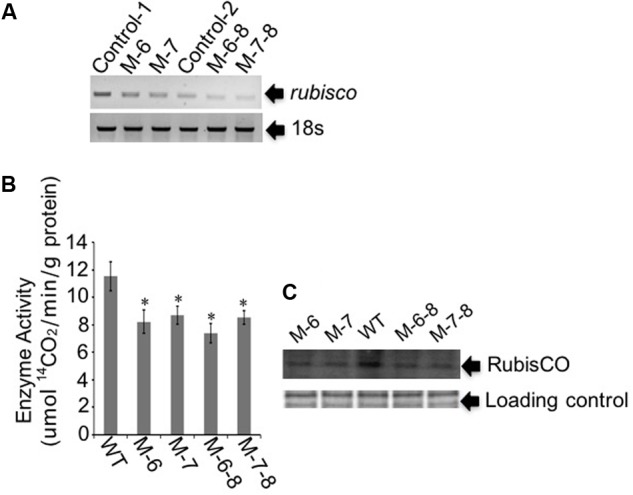
Gene expression and protein abundance analysis of the target RuBisCO gene. **(A)** Semi-quantitative RT-PCR analysis. In the single-deletion mutants M-6 and M-7, primers were designed for targeting *Ru-C*. In the double-deletion mutants M-6-8 and M-7-8, primers were designed for targeting *Ru-G*. Control-C, the control for targeting *Ru-C*; Control-G, the control for targeting *Ru-G*. 18S rDNA was used as the internal reference gene. **(B)** Measurement of the RuBisCO enzyme activity. The enzyme activity was defined as the amount of enzyme fixing ^14^CO_2_ in 1 min. Data represent the mean values ± SD of three individual experiments. Asterisks indicate significant differences between the control and mutants, as assessed by Student’s *T*-test (^∗^*p* < 0.05). **(C)** Western blot analysis of the RuBisCO protein. Top panel: total proteins were quantified and separated by SDS-PAGE, then used for the assay of RuBisCO protein. Bottom panel: Coomassie brilliant blue R250, used to stain the same proteins, was employed as loading control for western blotting.

The RuBisCO gene in non-photosynthetic *C. cohnii*, cultivated under heterotrophic conditions, is still transcribed (Sanchez-Puerta et al., 2007). Because the reactions involving RuBisCO consume energy and reducing equivalents (Dhingra et al., 2004; Leegood, 2007), we next aimed to redirect energy and reducing equivalents toward cell growth and lipid biosynthesis; for this, we constructed the RuBisCO-deleted mutants using the abovementioned transformation protocol for *C. cohnii*. Until now, the information about the structure and sequence of *C. cohnii* RuBisCO gene was lacking. However, studies on the dinoflagellate *Prorocentrum minimum* showed that over 10 transcribed units of the RuBisCO gene are present in the genome, and each transcribed unit contains four tandem copies of the RuBisCO coding region (1.46 kb each) interspersed by a 63-bp spacer. It was also estimated by real-time PCR analysis that each *P. minimum* genome harbors 148 ± 16 coding regions (Zhang and Lin, 2003). The sequence analysis of the coding regions revealed that the nucleotide sequences vary by approximately 1.0–9.0% among the coding regions; however, their inferred amino acid sequences are essentially identical (Zhang and Lin, 2003). Accordingly, we hypothesized that extensive gene duplication may also exist for the RuBisCO gene in *C. cohnii*. The RuBisCO gene has been found in the *C. cohnii* nuclear genome; this gene shares an evolutionary history with form II RuBisCO genes from peridinin-containing dinoflagellates (Sanchez-Puerta et al., 2007). Based on the sequence of the RuBisCO gene of *C. cohnii* (GenBank accession no. EB086308.1), we designed PCR primers (Ru-F and Ru-R) for amplification of RuBisCO gene fragments using both *C. cohnii* cDNA and genomic DNA as template, respectively. Two clear fragments of different sizes, designated as *Ru-C* and *Ru-G*, respectively, were obtained by PCR (**Supplementary Figure S4**). Sequencing and blast analysis showed that the *Ru-C* fragment matched to the reported *C. cohnii* RuBisCO gene, while the *Ru-G* fragment showed no significant similarity (data not shown). While we cannot rule out the possibility that *Ru-G* was from a different copy of the RuBisCO gene, we performed a phylogenetic analysis of this fragment with several known algal RuBisCO genes, including those from *Prorocentrum minimum*, *Karenia mikimotoi*, and *Symbiodinium* sp. The results showed that both *Ru-C* and *Ru-G* were clustered with other RuBisCO genes, suggesting that both could be putative RuBisCO genes in *C. cohnii* (**Supplementary Figure S5**). Obvious similarity was also observed when the DNA sequence of *Ru-G* was aligned against other RuBisCO genes from dinoflagellates and prokaryotes (**Supplementary Figure S6**). We first targeted the *Ru-C* fragment for the construction of a single-deletion mutant (construct see **Supplementary Figure S2B**). Three positive deletion clones (M-1, M-6, and M-7) were confirmed by growth patterns on plates with antibiotics (**Figure 3A**, top panel) and PCR analysis using a primer set of P-F and Hyg-R2 (**Figure 3A**, bottom panel). Using single-deletion mutants, we then targeted the *Ru-G* fragment for constructing double-deletion mutants (construct see **Supplementary Figure S2C**). The double-deletion mutants of both *Ru-C* and *Ru-G* were obtained and confirmed by double resistances and PCR analysis using a primer set of Ble-F3 and Ble-R2 (**Figure 3B**). In addition, to validate whether the resistance gene was successfully integrated into the genome, a PCR analysis with primers specific to RuBisCO gene and resistance gene yielded the expected amplification products in each of the mutants (i.e., primer sets of Hyg-F3 and Ru-R1, and Ble-F3 and Ru-R2, respectively) (**Supplementary Figure S7**). To further confirm the knockout of these two RuBisCO genes in *C. cohnii*, we also performed a RT-PCR analysis of the expression levels of the RuBisCO gene, ^14^C isotope-based enzymatic assay of RuBisCO activity, and western blotting analysis of the abundance of the RuBisCO protein in both WT and mutant cells. The semi-quantitative RT-PCR results showed that RuBisCO gene RNA expression was clearly decreased in the mutants (**Figure 4A**), and the RuBisCO enzymatic activity assay showed an approximately 30% decrease of the activity in all mutant cells (**Figure 4B**). These results suggest that the RuBisCO gene was transcribed and translated in *C. cohnii* under heterotrophic growth conditions, and the RuBisCO protein retained the specific carboxylation activity. In addition, the abundance of the RuBisCO protein was also clearly decreased in both single- and double-deletion mutants, as shown by western blotting (**Figure 4C**). Altogether, these data demonstrate that the knockdown of RuBisCO gene was evidenced in *C. cohnii*.

We found that the RuBisCO gene cannot be fully deleted. Two possible explanations are as follows. Copies of the RuBisCO gene are abundant in dinoflagellates (Zhang and Lin, 2003; Shi et al., 2013); currently, we were not able to delete all the RuBisCO genes due to the lack of the whole-genome sequence of *C. cohnii*. The mutants constructed were thus only knockdown mutants of the overall expression level of the RuBisCO genes in *C. cohnii*. It is also possible that RuBisCO is an essential gene at heterotrophic conditions; this notion is supported by the fact that the RuBisCO gene is transcribed and translated in *C. cohnii* under heterotrophic condition (Sanchez-Puerta et al., 2007). In *Ralstonia eutropha* H16, two RuBisCO genes in the CBB cycle are actually functional in CO_2_ fixation under heterotrophic conditions (Shimizu et al., 2013). Moreover, in *Rhodobacter sphaeroides* and *Rhodopseudomonas palustris*, the RuBisCO deletion mutants were lethal under malate-dependent photoheterotrophic conditions. This indicates the essential role of the CBB cycle in maintaining the redox balance in cells for efficient use of the carbon source (Laguna et al., 2011). Taken together, these results suggest that the RuBisCO gene may be essential for growth. In addition, RuBisCO can also function as an oxygenase involved in other metabolic activities such as glycine and serine biosynthesis (Wolfe and dePamphilis, 1998; Sekiguchi et al., 2002).

Although the activity of RuBisCO enzyme was decreased in both single- and double-deletion mutants, no significant decrease was observed in the double-deletion mutants when compared with the single-deletion mutants. This may be due to differential regulation, or roles, of the different RuBisCO genes under various growth conditions. For example, expression of a gene encoding a truncated large subunit of RuBisCO is salt-inducible in rice (Zhang et al., 1995), and an organ-specific expressed subunit of RuBisCO can alter the catalytic properties of the RuBisCO holoenzyme in rice (Morita et al., 2014).

### Comparative Growth and Lipid Content Analysis

**FIGURE 5:**
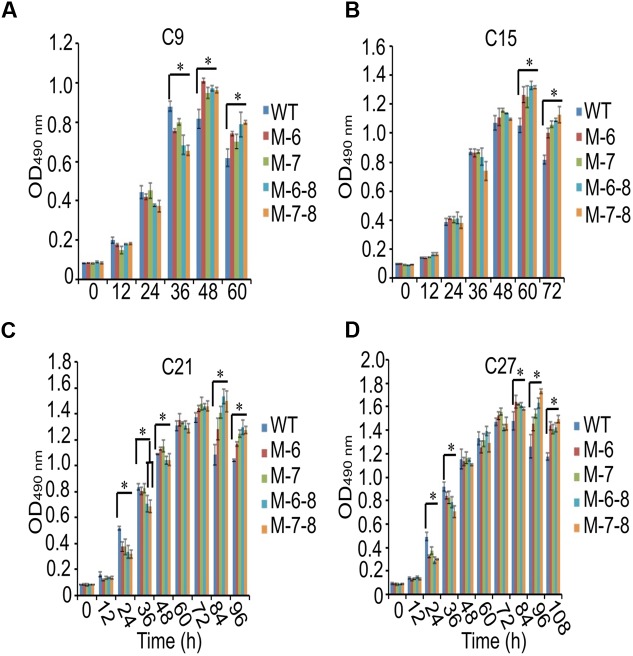
Effects of glucose concentration on growth of the RuBisCO mutants **(A–D)**. Data represent the mean values ± SD of three individual experiments **(A–D)**. Asterisks indicate significant differences between the control and mutants, as assessed by Student’s *T*-test (^∗^*p* < 0.05). **(A–D)** C9, C15, C21, and C27 represent 9, 15, 21, and 27 g/L glucose added into the culture media, respectively.

**FIGURE 6:**
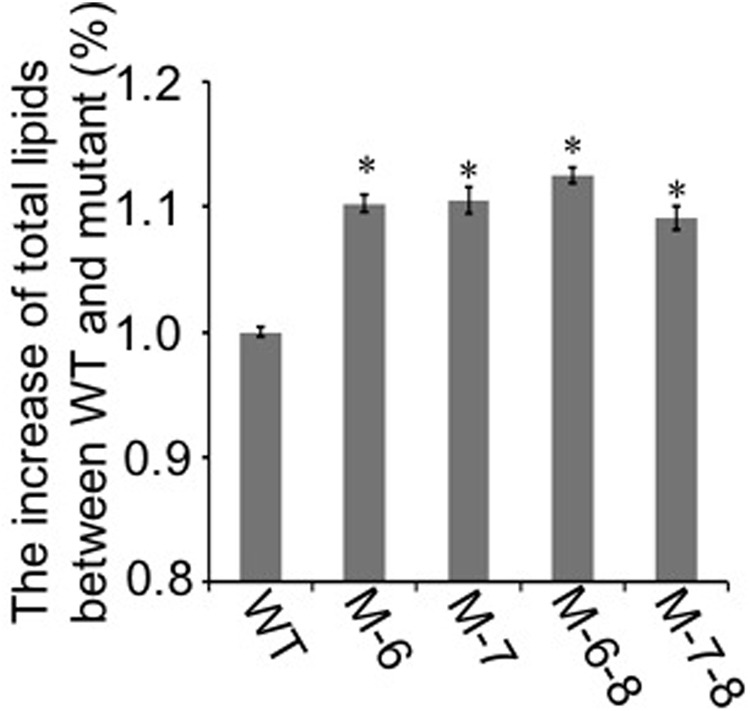
Lipid accumulation in the RuBisCO-deletion mutants and wild-type. The lipid content in the control was set as 100%, and percentage changes in the mutants are shown. Data represent the mean ± SD of three independent experiments. Asterisks indicate significant differences between the control and mutants, as evaluated by Student’s *T*-test (^∗^*p* < 0.05).

To demonstrate that the knockout of the RuBisCO gene was metabolically advantageous to *C. cohnii* cells, we conducted a comparative growth analysis of the RuBisCO mutants and wild-type; this analysis was performed on basal media with a glucose concentration ranging from 9 to 27 g/L. As shown in **Figure 5**, the deletion of the RuBisCO gene in *C. cohnii* resulted in enhanced cellular growth. Growth increase was more significant at a high glucose concentration of 21 g/L than at the basal concentration of 9 g/L. However, when glucose concentration reached 27 g/L, enhancement of growth in the mutants became less significant (**Figure 5D**). Under this condition, although the growth of the mutants was slightly slow at early exponential phase, it caught up and reached higher levels at the stationary phase (**Figure 5C**). Growth increases in the RuBisCO-deleted *C. cohnii* were also confirmed by statistical analysis with *p*-values less than 0.05; the results showed that at the late growth phase (i.e., stationary phase), the accumulated growth differences were more significant at all the tested concentrations of glucose. We also examined lipid accumulation in the wild-type and in RuBisCO-deleted mutants during the late growth phase, when the most significant increase in cell growth was observed (**Figure 5C**). Cells were harvested at 84 h and extracted for total lipids. We observed a significant increase in lipid accumulation, with an average improvement of approximately 10.6% (**Figure 6**).

### Targeted Metabolomic Analysis

We next explored the possible molecular mechanisms contributing to the enhanced growth and lipid accumulation in RuBisCO-deleted mutants. For this, we used LC-MS-based targeted metabolomics to quantify the time-series changes of 24 selected metabolites related to central carbohydrate and energy metabolism in *C. cohnii* cells. Using the optimized protocol described previously (Sui et al., 2014; Li et al., 2015), we achieved reproducible analysis of 24 selected intracellular metabolites for *C. cohnii*. For the metabolomic analysis, two single-deletion and two double-deletion RuBisCO mutants, and wild-type *C. cohnii*, were selected and cultivated for 4 days in a medium with 21 g/L glucose. Cell samples were collected at 48 and 84 h, which corresponds to exponential and stationary growth phases, respectively. The time-series changes in targeted metabolites were determined using the LC-MS approach (raw metabolomic data are provided in **Supplementary Table S2**). For each sample, three biological replicates were prepared and collected separately, and each of the biological replicates was analyzed twice by LC-MS.

**FIGURE 7:**
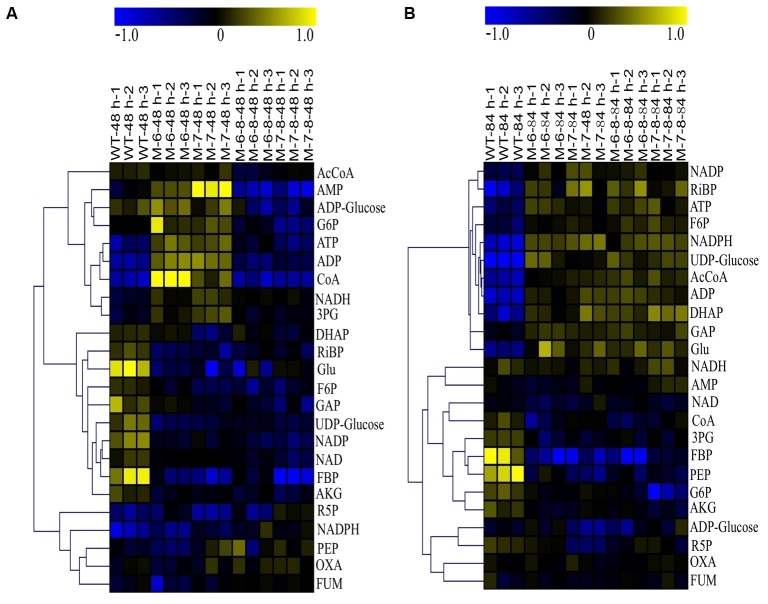
Heatmaps of LC-MS metabolomic profiles. **(A)** 48 h; **(B)** 84 h. The color in the heatmap indicates log2 transformed ratio of a given metabolite versus average concentration of the metabolites in all samples.

The quality of the LC-MS metabolomics analysis was confirmed by a principal component analysis (PCA) (**Supplementary Figure S8**). Moreover, mutant cells were at the farthest distance from the controls at 84 h, as shown in **Supplementary Figure S8B**; this indicates the most significant metabolic changes in cells at this time point, consistent with their growth patterns (**Figure 5C**). To better interpret the qualitative information of the results, we generated heatmaps of the two time points using MultiExperiment Viewer software. Similar methodology has been successfully applied to analyze transcriptomic data previously (Putri et al., 2013), in which the ratio between the abundance of the metabolite under a given condition, and the average abundance of the metabolite in all samples, was calculated for each metabolite. The analysis showed that at 48 h, changes in the metabolites were detectable for single-deletion mutants. However, the fold changes were relatively small. The changes were not obvious for the double-deletion mutants, which was consistent with their growth patterns. At 48 h, most of the detected metabolites were downregulated, except for ADP-Glucose, G6P, ATP, ADP, CoA, NADH, and AMP in the RuBisCO single-deletion mutants. Nearly all metabolites were downregulated in the double-deletion mutants compared with the wild-type (**Figure 7A**). At 84 h when the most significant differential growth was recorded (**Figure 5C**), we observed upregulation of most metabolites in both single- and double-deletion mutants; these metabolites included F6P, NADP, NADPH, UDP-Glucose, Ac-CoA, ADP, ATP, RiBP, DHAP, GAP, and Glu (**Figure 7B**). Thus, we hypothesized that deletion of the RuBisCO gene in the CBB cycle may disrupt the redox balance in the plastid, thereby enhancing the central carbohydrate and energy metabolism to improve cell growth and lipid accumulation.

These results showed an upregulation of NADH and downregulation of NAD, which increased the ratio of NADH/NAD at 48 h in the single-deletion mutant (**Figure 7A**). The high ratio of NADH/NAD provides a reducing environment, beneficial to cell growth (Schwartz et al., 1974). However, at 84 h, the ratio of NADH/NAD in the wild-type and mutants had barely changed, and the content of Ac-CoA, ATP, and NADPH were improved; this indicates that multiple mechanisms may be involved at different growth phases. For example, the metabolite Ac-CoA can induce cell growth and proliferation by promoting the acetylation of histones at growth genes (Cai et al., 2011). Increasing the content of intracellular NADPH by improving the activity of glucose-6-phosphate dehydrogenase also stimulates cell growth (Stanton, 2012). In addition, AKG can behave as an inhibitor of malic enzyme, which is considered a key pathway for the generation of NADPH (Gupta et al., 2013). Therefore, the decreased levels of AKG in the mutants may result in reduced inhibition of the generation of NADPH. The downregulation of AKG was consistent with the results of previous studies (Ratledge, 2002; Sui et al., 2014), showing that a decrease in AKG releases inhibition of NADPH biosynthesis, leading to increased fatty acid biosynthesis (Ratledge, 2002). Moreover, the energy currency ATP was upregulated, which is beneficial for efficient transcription and protein synthesis (Gaal et al., 1997). This is consistent with a study on *Escherichia coli*, showing that intracellular ATP concentration was found to increase proportionally with the growth rate (Schneider and Gourse, 2004). Moreover, when cells enter the phase in which they accumulate lipids, the pentose phosphate pathway (PPP) is downregulated (Sui et al., 2014; Wase et al., 2014; Li et al., 2015); our analysis consistently showed that R5P was downregulated.

Because our metabolomics analysis indicated the upregulated energy metabolism as one of the possible reasons for enhanced growth in the RuBisCO-deleted mutants, we next validated these results using quantitative RT-PCR. For this, we compared the expression levels of two selected genes, ATP synthase and ribosome coding genes, in wild-type and mutant cells. We hypothesized that if cells can retain a high growth rate, the expression level of ATP synthase and ribosomes must be increased to maintain a high-level supply of ATP and proteins. The results show that both genes were significantly upregulated in the mutants (**Supplementary Figure S9**); this is consistent with enhanced energy metabolism, as shown by the metabolomics analysis. In the CBB cycle, one molecular CO_2_ fixation consumes 3 molecular ATPs and 2 molecular NADPH (Alric et al., 2010). If the RuBisCO gene is deleted, more ATP and NADPH is saved (Laguna et al., 2011). These results support our hypothesis that the deletion or knockdown of the highly abundant, but unnecessary gene, RuBisCO in the CBB cycle may disrupt the redox balance in the plastid. This is beneficial for re-directing the carbon and energy to cell growth and lipid accumulation under conditions of heterotrophic growth. However, the molecular mechanism of how redox unbalancing in the RuBisCO-deleted mutant enhances the central carbohydrate and energy metabolism needs to be further elucidated.

## Author Contributions

JD, LC, and WZ designed the research. JD and XS performed the molecular biology experiments. XZ performed the LC-MS analysis. JD and WZ drafted and revised the manuscript. All authors read and approved the final manuscript.

## Conflict of Interest Statement

The authors declare that the research was conducted in the absence of any commercial or financial relationships that could be construed as a potential conflict of interest.

## Supplementary Material

**FIGURE S1 |** Antibiotic sensitivity tests of *C. cohnii*. **(A)** Hygromycin; **(B)** bleomycin.

**FIGURE S2 |** Scheme of transformant construction. **(A–C)** C1: 18S-HR; C2: Ru-C-HR; C3: Ru-G-HR. Each construct consists of a left flank and a right flank, separated by the selection marker. Primer positions (arrows) for the PCR analysis of the mutants are indicated.

**FIGURE S3 |** Flow chart of the electroporation transformation protocol.

**FIGURE S4 |** PCR cloning of RuBisCO gene. Amplifying the RuBisCO fragment. **(Left)** PCR was conducted using cDNA of *C. cohnii* as template. M, DNA marker III. **(Right)** genomic DNA of *C. cohnii* was used as template.

**FIGURE S5 |** Neighbor-joining (NJ)-based phylogenetic analysis of Ru-C (*C. cohnii-1*), Ru-G (*C. cohnii-2*), and RuBisCO. This phylogenetic analysis was based on nucleotide sequences, and Clustal X and MEGA4 were utilized to produce the phylogenetic tree.

**FIGURE S6 |** Sequence alignment of *Ru-G* gene against other RuBisCO gene in dinoflagellates and prokaryotes. GenBank numbers of RuBisCO genes from *Symbiodinium sp*. and *Hydrogenovibrio marinusis* are AF298221.1 and D28135.1, respectively.

**FIGURE S7 |** PCR validation of the RuBisCO mutants. Genome DNA was amplified by PCR, with the primer pairs specific to RuBisCO genes and resistance genes. **(A)** Detection of *hygR* and *Ru-C* downstream; **(B)** Detection of *Ru-G* and *Ble*. H_2_O was used as negative control to exclude contamination of the PCR reagents.

**FIGURE S8 |** Score plots of PCA analysis of metabolomic profiling. **(A)** 48 h; **(B)** 84 h. For the PCA plots, PC1 and PC2 represent the 1st and 2nd components of the PCA analysis, respectively; *R*^2^ represents the sum of the square captured by the model.

**FIGURE S9 |** RT-qPCR analysis of ATP synthase and ribosome encoding genes. Cells were cultivated to 84 h in basal medium with 21 g/L glucose, and then harvested for RNA extraction. **(A)** ATP synthase; **(B)** ribosome. Data represent the mean ± SD of three individual experiments. Asterisks indicate significant differences between the mutants and wild-type as evaluated by Student’s *T*-test (^∗^*p* < 0.05).

**TABLE S1 |** Primers used in this study.

**TABLE S2 |** LC-MS metabolomic dataset of *C. cohnii*^∗^.

## References

[B1] Agostoni C. Riva E. Trojan S. Bellu R. Giovannini M. (1995). Docosahexaenoic acid status and developmental quotient of healthy term infants. *Lancet* 346:638 10.1016/S0140-6736(95)91469-2 7651024

[B2] Alric J. Lavergne J. Rappaport F. (2010). Redox and ATP control of photosynthetic cyclic electron flow in *Chlamydomonas reinhardtii* (I) aerobic conditions. *Biochim. Biophys. Acta* 1797 44–51. 10.1016/j.bbabio.2009.07.009 19651104

[B3] Avidan O. Brandis A. Rogachev I. Pick U. (2015). Enhanced acetyl-CoA production is associated with increased triglyceride accumulation in the green alga *Chlorella desiccata*. *J. Exp. Bot.* 66 3725–3735. 10.1093/jxb/erv166 25922486 PMC4473976

[B4] Barclay W. R. Meager K. M. Abril J. R. (1994). Heterotrophic production of long chain omega-3 fatty acids utilizing algae and algae-like microorganisms. *J. Appl. Phycol.* 6 123–129. 10.1007/BF02186066

[B5] Bell M. V. Henderson R. J. (1990). Molecular species composition of phosphatidylcholine from *Crypthecodinium cohnii* in relation to growth temperature. *Lipids* 25 115–118. 10.1007/BF02562215

[B6] Cai L. Sutter B. M. Li B. Tu B. P. (2011). Acetyl-CoA induces cell growth and proliferation by promoting the acetylation of histones at growth genes. *Mol. Cell* 42 426–437. 10.1016/j.molcel.2011.05.004 21596309 PMC3109073

[B7] Carlson S. E. (1996). Arachidonic acid status of human infants: influence of gestational age at birth and diets with very long chain n-3 and n-6 fatty acids. *J. Nutr.* 126 1092S–1098S 10.1093/jn/126.suppl_4.1092S 8642439

[B8] Cheng R. -B. Lin X. -Z. Wang Z. -K. Yang S. -J. Rong H. Ma Y. (2010). Establishment of a transgene expression system for the marine microalga *Schizochytrium* by 18S rDNA-targeted homologous recombination. *World J. Microbiol. Biotechnol.* 27 737–741. 10.1007/s11274-010-0510-8

[B9] de Swaaf M. E. de Rijk T. C. Eggink G. Sijtsma L. (1999). Optimisation of docosahexaenoic acid production in batch cultivations by *Crypthecodinium cohnii*. *J. Biotechnol.* 70 185–192. 10.1016/S0168-1656(99)00071-1

[B10] de Swaaf M. E. Pronk J. T. Sijtsma L. (2003). Fed-batch cultivation of the docosahexaenoic-acid-producing marine alga *Crypthecodinium cohnii* on ethanol. *Appl. Microbiol. Biotechnol.* 61 40–43. 12658513 10.1007/s00253-002-1118-1

[B11] De Swaaf M. E. Sijtsma L. Pronk J. T. (2003). High-cell-density fed-batch cultivation of the docosahexaenoic acid producing marine alga *Crypthecodinium cohnii*. *Biotechnol. Bioeng.* 81 666–672. 12529880 10.1002/bit.10513

[B12] Dhingra A. Portis A. R. Jr. Daniell H. (2004). Enhanced translation of a chloroplast-expressed RbcS gene restores small subunit levels and photosynthesis in nuclear RbcS antisense plants. *Proc. Natl. Acad. Sci. U.S.A.* 101 6315–6320. 10.1073/pnas.0400981101 15067115 PMC395966

[B13] Gaal T. Bartlett M. S. Ross W. Turnbough C. L. Jr. Gourse R. L. (1997). Transcription regulation by initiating NTP concentration: rRNA synthesis in bacteria. *Science* 278 2092–2097. 10.1126/science.278.5346.2092 9405339

[B14] Gupta V. Thakur R. S. Reddy C. R. K. Jha B. (2013). Central metabolic processes of marine macrophytic algae revealed from NMR based metabolome analysis. *RSC Adv.* 3 7037–7047. 10.1039/c3ra23017a

[B15] Hajdukiewicz P. Svab Z. Maliga P. (1994). The small, versatile pPZP family of *Agrobacterium* binary vectors for plant transformation. *Plant Mol. Biol.* 25 989–994. 10.1007/BF00014672 7919218

[B16] Hamilton M. L. Haslam R. P. Napier J. A. Sayanova O. (2014). Metabolic engineering of *Phaeodactylum tricornutum* for the enhanced accumulation of omega-3 long chain polyunsaturated fatty acids. *Metab. Eng.* 22 3–9. 10.1016/j.ymben.2013.12.003 24333273 PMC3985434

[B17] Harrington G. W. Holz G. G. Jr. (1968). The monoenoic and docosahexaenoic fatty acids of a heterotrophic dinoflagellate. *Biochim. Biophys. Acta* 164 137–139. 10.1016/0005-2760(68)90083-0 5680291

[B18] Hornstra G. (2000). Essential fatty acids in mothers and their neonates. *Am. J. Clin. Nutr.* 71 1262S–1269S. 10.1093/ajcn/71.5.1262s 10799400

[B19] Iwai M. Ikeda K. Shimojima M. Ohta H. (2014). Enhancement of extraplastidic oil synthesis in *Chlamydomonas reinhardtii* using a type-2 diacylglycerol acyltransferase with a phosphorus starvation-inducible promoter. *Plant Biotechnol. J.* 12 808–819. 10.1111/pbi.12210 24909748 PMC4160818

[B20] Jeon K. Suresh A. Kim Y. -C. (2013). Highly efficient molecular delivery into *Chlamydomonas reinhardtii* by electroporation. *Korean J. Chem. Eng.* 30 1626–1630. 10.1007/s11814-013-0098-4

[B21] Jiang Y. Chen F. Liang S. Z. (1999). Production potential of docosahexaenoic acid by the heterotrophic marine dinoflagellate *Crypthecodinium cohnii*. *Process Biochem.* 34 633–637. 10.1016/S0032-9592(98)00134-4 12658513

[B22] Komatsu M. Uchiyama T. Omura S. Cane D. E. Ikeda H. (2010). Genome-minimized *Streptomyces* host for the heterologous expression of secondary metabolism. *Proc. Natl. Acad. Sci. U.S.A.* 107 2646–2651. 10.1073/pnas.0914833107 20133795 PMC2823899

[B23] Kuratko C. N. Salem N. (2013). Docosahexaenoic acid from algal oil. *Eur. J. Lipid Sci. Technol.* 115 965–976. 10.1002/ejlt.201300060

[B24] Kwok A. C. Mak C. C. Wong F. T. Wong J. T. (2007). Novel method for preparing spheroplasts from cells with an internal cellulosic cell wall. *Eukaryot. Cell* 6 563–567. 10.1128/EC.00301-06 17259549 PMC1828928

[B25] Laguna R. Tabita F. R. Alber B. E. (2011). Acetate-dependent photoheterotrophic growth and the differential requirement for the Calvin-Benson-Bassham reductive pentose phosphate cycle in *Rhodobacter sphaeroides* and *Rhodopseudomonas palustris*. *Arch. Microbiol.* 193 151–154. 10.1007/s00203-010-0652-y 21104179

[B26] Leegood R. C. (2007). A welcome diversion from photorespiration. *Nat. Biotechnol.* 25 539–540. 10.1038/nbt0507-539 17483837

[B27] Li J. Niu X. Pei G. Sui X. Zhang X. Chen L. (2015). Identification and metabolomic analysis of chemical modulators for lipid accumulation in *Crypthecodinium cohnii*. *Bioresour. Technol.* 191 362–368. 10.1016/j.biortech.2015.03.068 25818259

[B28] Lilley R. M. Ralph P. J. Larkum A. W. (2010). The determination of activity of the enzyme Rubisco in cell extracts of the dinoflagellate alga *Symbiodinium* sp. by manganese chemiluminescence and its response to short-term thermal stress of the alga. *Plant Cell Environ.* 33 995–1004. 10.1111/j.1365-3040.2010.02121.x 20102538

[B29] Livak K. J. Schmittgen T. D. (2001). Analysis of relative gene expression data using real-time quantitative PCR and the 2^-ΔΔ*C*_T_^ Method. *Methods* 25 402–408. 10.1006/meth.2001.1262 11846609

[B30] Lorimer G. H. Badger M. R. Andrews T. J. (1977). D-Ribulose-1,5-bisphosphate carboxylase-oxygenase: improved methods for the activation and assay of catalytic activities. *Anal. Biochem.* 78 66–75. 10.1016/0003-2697(77)90009-4 848758

[B31] Lumbreras V. Stevens D. R. Purton S. (1998). Efficient foreign gene expression in *Chlamydomonas reinhardtii* mediated by an endogenous intron. *Plant J.* 14 441–447. 10.1046/j.1365-313X.1998.00145.x

[B32] Morita K. Hatanaka T. Misoo S. Fukayama H. (2014). Unusual small subunit that is not expressed in photosynthetic cells alters the catalytic properties of Rubisco in rice. *Plant Physiol.* 164 69–79. 10.1104/pp.113.228015 24254313 PMC3875826

[B33] Pisabarro A. Correia A. Martin J. F. (1998). Characterization of the rrnB operon of the plant pathogen *Rhodococcus fascians* and targeted integrations of exogenous genes at rrn loci. *Appl. Microbiol. Biotechnol.* 64 1276–1282. 9546162 10.1128/aem.64.4.1276-1282.1998 PMC106141

[B34] Putri S. P. Nakayama Y. Matsuda F. Uchikata T. Kobayashi S. Matsubara A. (2013). Current metabolomics: practical applications. *J. Biosci. Bioeng.* 115 579–589. 10.1016/j.jbiosc.2012.12.007 23369275

[B35] Qiao K. Wasylenko T. M. Zhou K. Xu P. Stephanopoulos G. (2017). Lipid production in *Yarrowia lipolytica* is maximized by engineering cytosolic redox metabolism. *Nat. Biotechnol.* 35 173–177. 10.1038/nbt.3763 28092657

[B36] Radakovits R. Eduafo P. M. Posewitz M. C. (2011). Genetic engineering of fatty acid chain length in *Phaeodactylum tricornutum*. *Metab. Eng.* 13 89–95. 10.1016/j.ymben.2010.10.003 20971205

[B37] Ratledge C. (2002). Regulation of lipid accumulation in oleaginous micro-organisms. *Biochem. Soc. Trans.* 30 1047–1050. 10.1042/bst0301047 12440969

[B38] Ratledge C. (2004). Fatty acid biosynthesis in microorganisms being used for Single Cell Oil production. *Biochimie* 86 807–815. 10.1016/j.biochi.2004.09.017 15589690

[B39] Richard J. P. Melikov K. Vives E. Ramos C. Verbeure B. Gait M. J. (2003). Cell-penetrating peptides: a reevaluation of the mechanism of cellular uptake. *J. Biol. Chem.* 278 585–590. 10.1074/jbc.M209548200 12411431

[B40] Sanchez-Puerta M. V. Lippmeier J. C. Apt K. E. Delwiche C. F. (2007). Plastid genes in a non-photosynthetic dinoflagellate. *Protist* 158 105–117. 10.1016/j.protis.2006.09.004 17150410

[B41] Schneider D. A. Gourse R. L. (2004). Relationship between growth rate and ATP concentration in *Escherichia coli*: a bioassay for available cellular ATP. *J. Biol. Chem.* 279 8262–8268. 10.1074/jbc.M311996200 14670952

[B42] Schwartz J. P. Passonneau J. V. Johnson G. S. Pastan I. (1974). The effect of growth conditions on NAD+ and NADH concentrations and the NAD^+^:NADH ratio in normal and transformed fibroblasts. *J. Biol. Chem.* 249 4138–4143. 4369293

[B43] Sekiguchi H. Moriya M. Nakayama T. Inouye I. (2002). Vestigial chloroplasts in heterotrophic stramenopiles *Pteridomonas danica* and *Ciliophrys infusionum* (Dictyochophyceae). *Protist* 153 157–167. 10.1078/1434-4610-00094 12125757

[B44] Shi X. Zhang H. Lin S. (2013). Tandem repeats, high copy number and remarkable diel expression rhythm of form II RuBisCO in *Prorocentrum donghaiense* (Dinophyceae). *PLoS One* 8:e71232. 10.1371/journal.pone.0071232 23976999 PMC3747160

[B45] Shimizu R. Chou K. Orita I. Suzuki Y. Nakamura S. Fukui T. (2013). Detection of phase-dependent transcriptomic changes and Rubisco-mediated CO2 fixation into poly (3-hydroxybutyrate) under heterotrophic condition in *Ralstonia eutropha* H16 based on RNA-seq and gene deletion analyses. *BMC Microbiol.* 13:169. 10.1186/1471-2180-13-169 23879744 PMC3734047

[B46] Sijtsma L. de Swaaf M. E. (2004). Biotechnological production and applications of the omega-3 polyunsaturated fatty acid docosahexaenoic acid. *Appl. Microbiol. Biotechnol.* 64 146–153. 10.1007/s00253-003-1525-y 14740186

[B47] Song X. Diao J. Ji J. Wang G. Li Z. Wu J. (2016). Overexpression of lycopene epsilon-cyclase gene from lycium chinense confers tolerance to chilling stress in *Arabidopsis thaliana*. *Gene* 576 395–403. 10.1016/j.gene.2015.10.051 26526130

[B48] Stanton R. C. (2012). Glucose-6-phosphate dehydrogenase, NADPH, and cell survival. *IUBMB Life* 64 362–369. 10.1002/iub.1017 22431005 PMC3325335

[B49] Sudhani H. P. García-Murria M. J. Marín-Navarro J. García-Ferris C. Peñarrubia L. Moreno J. (2015). Assay of the carboxylase activity of Rubisco from *Chlamydomonas reinhardtii*. *Bio Protoc.* 5:e1672 10.21769/BioProtoc.1672

[B50] Sui X. Niu X. Shi M. Pei G. Li J. Chen L. (2014). Metabolomic analysis reveals mechanism of antioxidant butylated hydroxyanisole on lipid accumulation in *Crypthecodinium cohnii*. *J. Agric. Food Chem.* 62 12477–12484. 10.1021/jf503671m 25436856

[B51] Sun Y. Yang Z. Gao X. Li Q. Zhang Q. Xu Z. (2005). Expression of foreign genes in *Dunaliella* by electroporation. *Mol. Biotechnol.* 30 185–192. 10.1385/MB:30:3:185 15988044

[B52] Te M. R. Miller D. J. (1998). Genetic transformation of dinoflagellates (*Amphidinium* and *Symbiodinium*): expression of GUS in microalgae using heterologous promoter constructs. *Plant J.* 13 427–435. 10.1046/j.1365-313X.1998.00040.x

[B53] Wang H. L. Postier B. L. Burnap R. L. (2002). Optimization of fusion PCR for in vitro construction of gene knockout fragments. *Biotechniques* 33 26 28 30 passim. 12139251 10.2144/02331bm02

[B54] Wang X. Liu W. Xin C. Zheng Y. Cheng Y. Sun S. (2016). Enhanced limonene production in cyanobacteria reveals photosynthesis limitations. *Proc. Natl. Acad. Sci. U.S.A.* 113 14225–14230. 10.1073/pnas.1613340113 27911807 PMC5167140

[B55] Wase N. Black P. N. Stanley B. A. Dirusso C. C. (2014). Integrated quantitative analysis of nitrogen stress response in *Chlamydomonas reinhardtii* using metabolite and protein profiling. *J. Proteome Res.* 13 1373–1396. 10.1021/pr400952z 24528286

[B56] Weeks D. P. (2011). Homologous recombination in *Nannochloropsis*: a powerful tool in an industrially relevant alga. *Proc. Natl. Acad. Sci. U.S.A.* 108 20859–20860. 10.1073/pnas.1118670109 22184230 PMC3248476

[B57] Weete J. D. Kim H. Gandhi S. R. Wang Y. Dute R. (1997). Lipids and ultrastructure of *Thraustochytrium* sp. ATCC 26185. *Lipids* 32 839–845. 10.1007/s11745-997-0107-z 9270975

[B58] Wolfe A. D. dePamphilis C. W. (1998). The effect of relaxed functional constraints on the photosynthetic gene rbcL in photosynthetic and nonphotosynthetic parasitic plants. *Mol. Biol. Evol.* 15 1243–1258. 10.1093/oxfordjournals.molbev.a025853 9787431

[B59] Yan J. Cheng R. Lin X. You S. Li K. Rong H. (2013). Overexpression of acetyl-CoA synthetase increased the biomass and fatty acid proportion in microalga *Schizochytrium*. *Appl. Microbiol. Biotechnol.* 97 1933–1939. 10.1007/s00253-012-4481-6 23070649

[B60] Zhang H. Lin S. (2003). Complex gene structure of the form II Rubisco in the dinoflagellate *Prorocentrum minimum* (dinophyceae). *J.Phycol.* 39 1160–1171. 10.1111/j.0022-3646.2003.03-055.x

[B61] Zhang J.-S. Gu J. Liu F.-H. Chen S.-Y. (1995). A gene encoding a truncated large subunit of Rubisco is transcribed and salt-inducible in rice. *Theor. Appl. Genet.* 91 361–366. 10.1007/BF00220900 24169786

