# Supplementary figures and images for: Genetic Engineering of Crypthecodinium cohnii to Increase Growth and Lipid Accumulation
Source: Front Microbiol. 2018 Mar 19;9:492. doi: 10.3389/fmicb.2018.00492 (PMC5868476; doi:10.3389/fmicb.2018.00492)

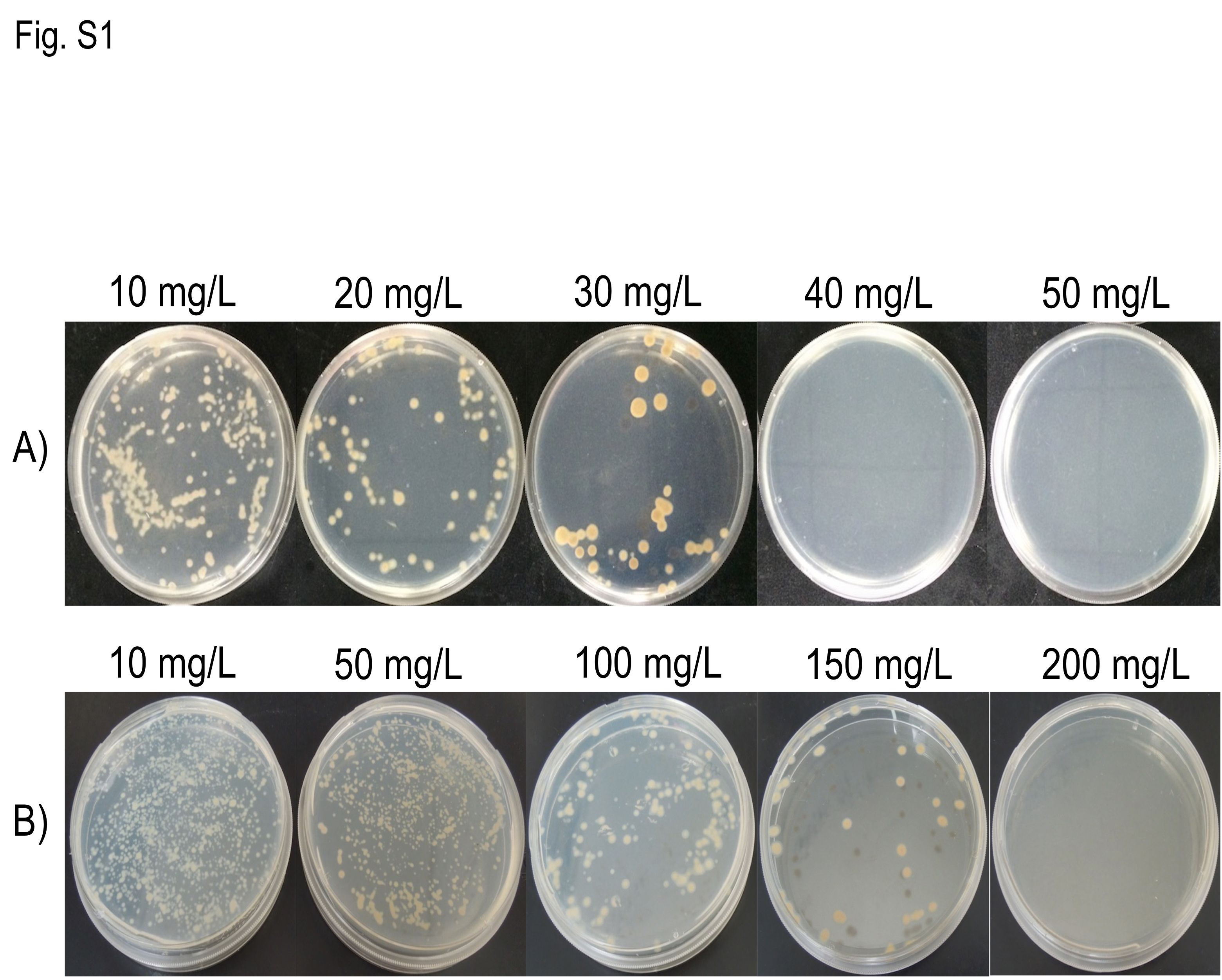

Supplement: Supplementary file 1 [file Image_1.JPEG]

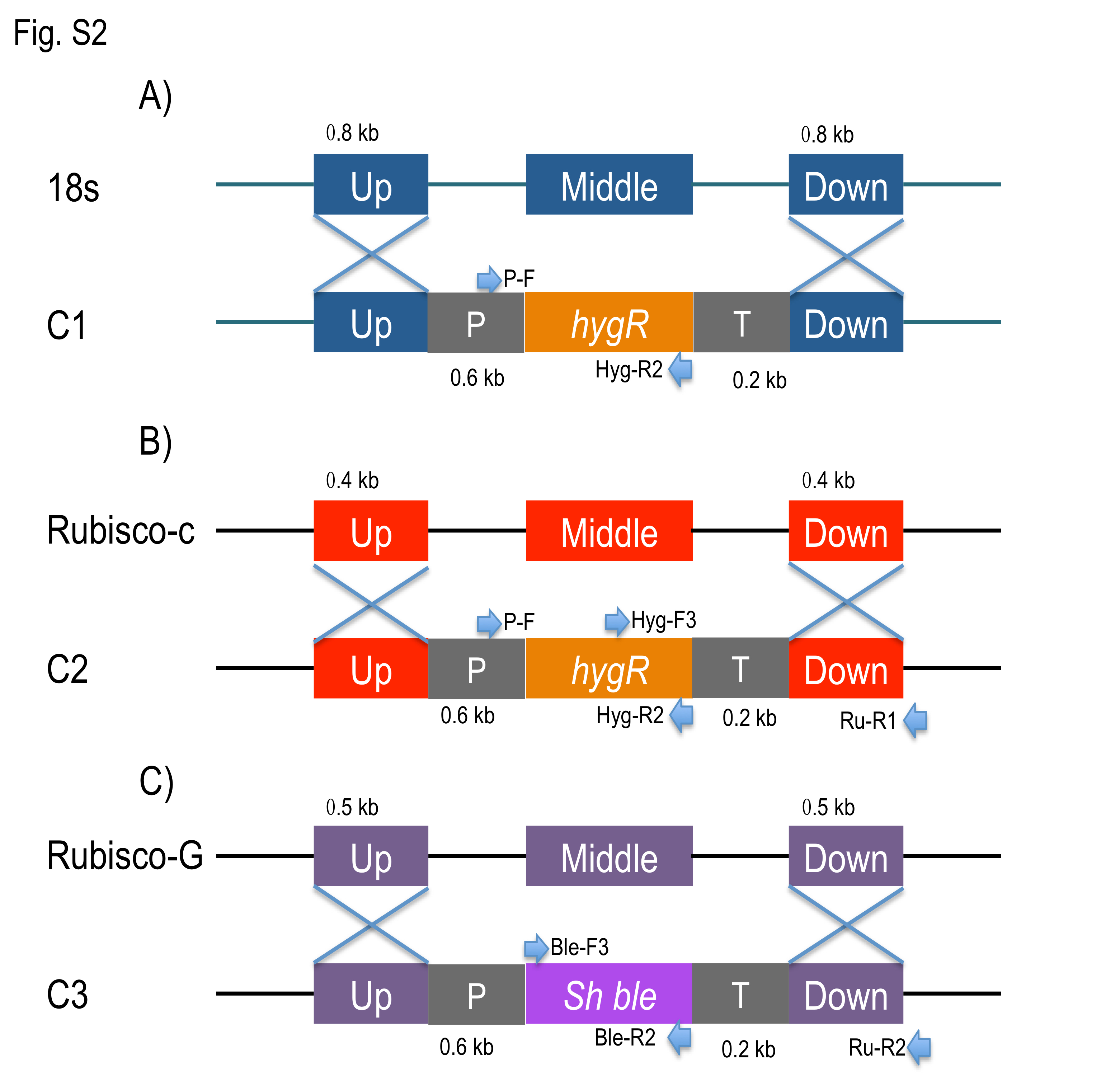

Supplement: Supplementary file 2 [file Image_2.JPEG]

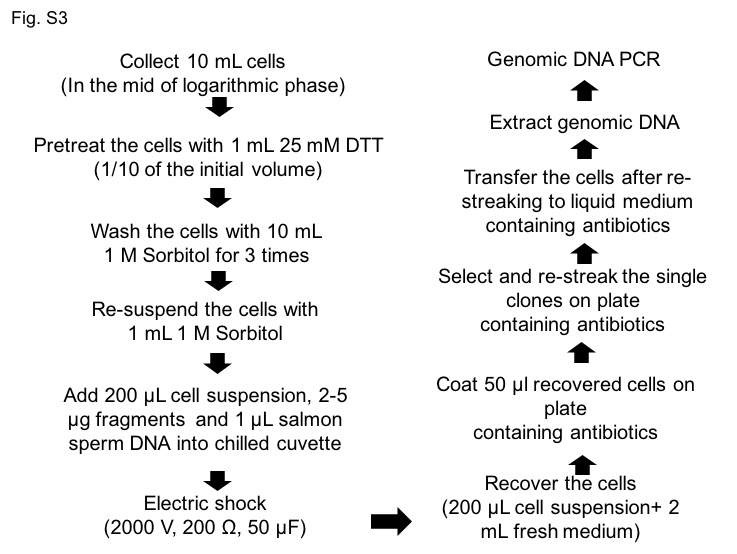

Supplement: Supplementary file 3 [file Image_3.JPEG]

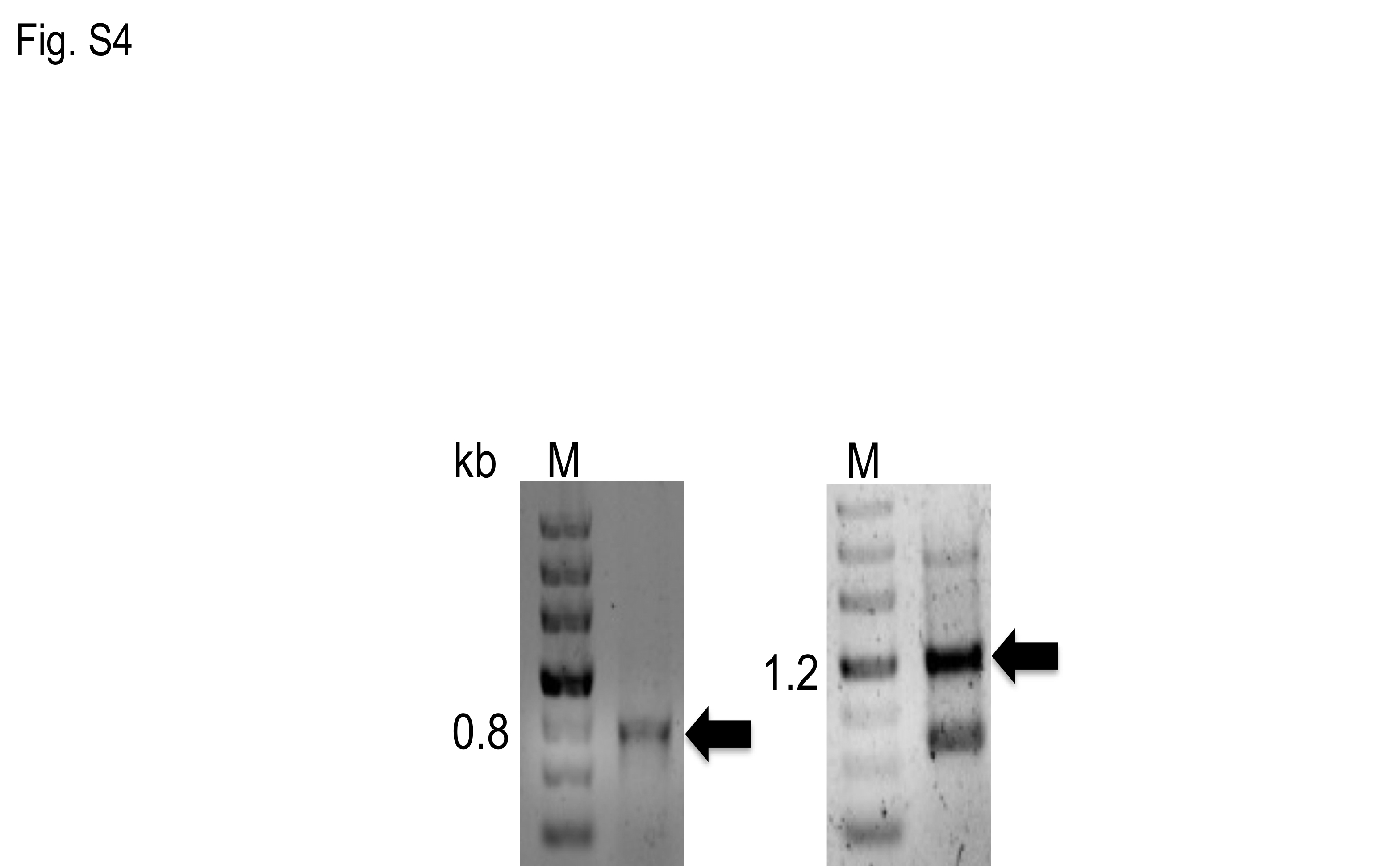

Supplement: Supplementary file 4 [file Image_4.JPEG]

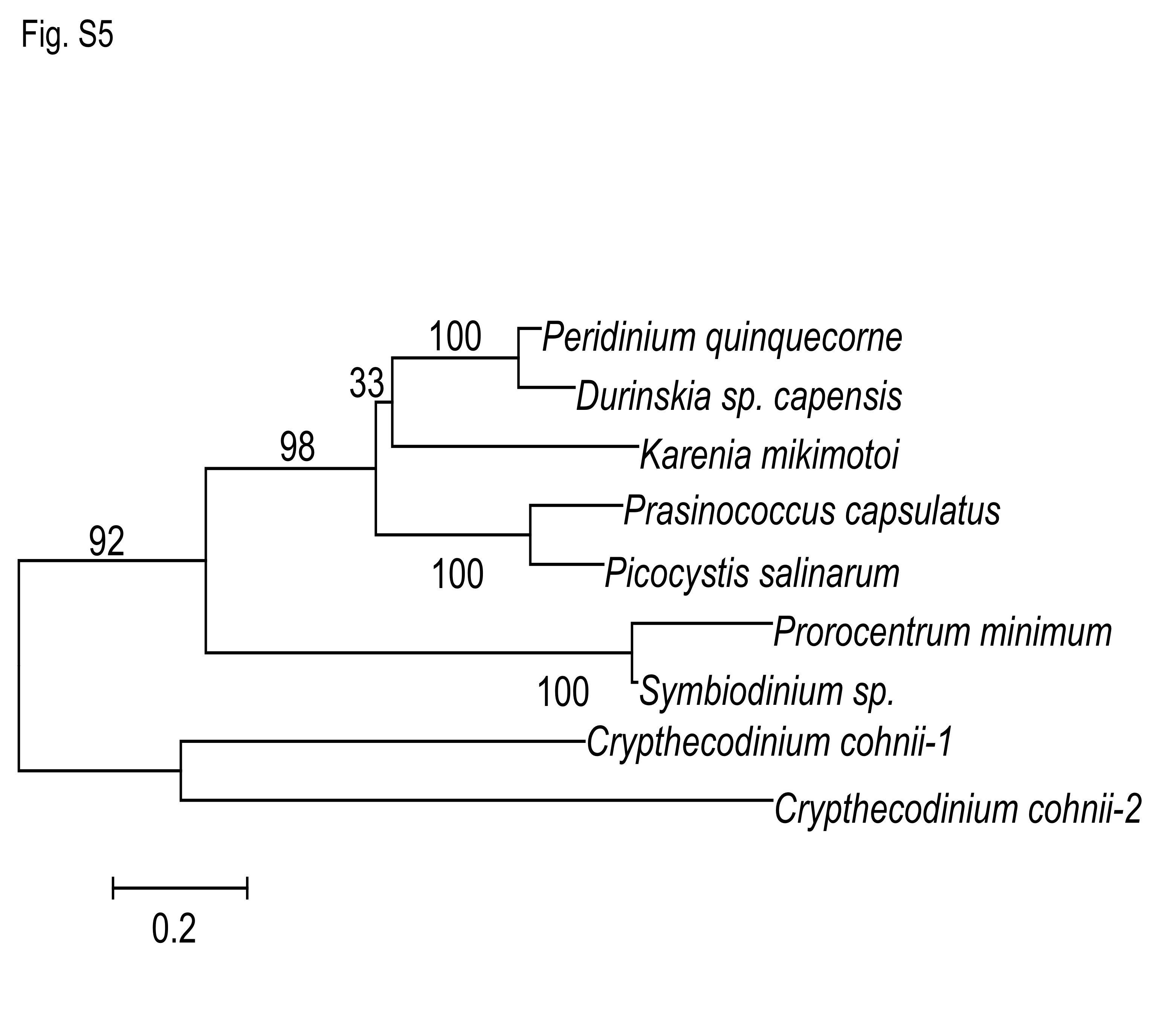

Supplement: Supplementary file 5 [file Image_5.JPEG]

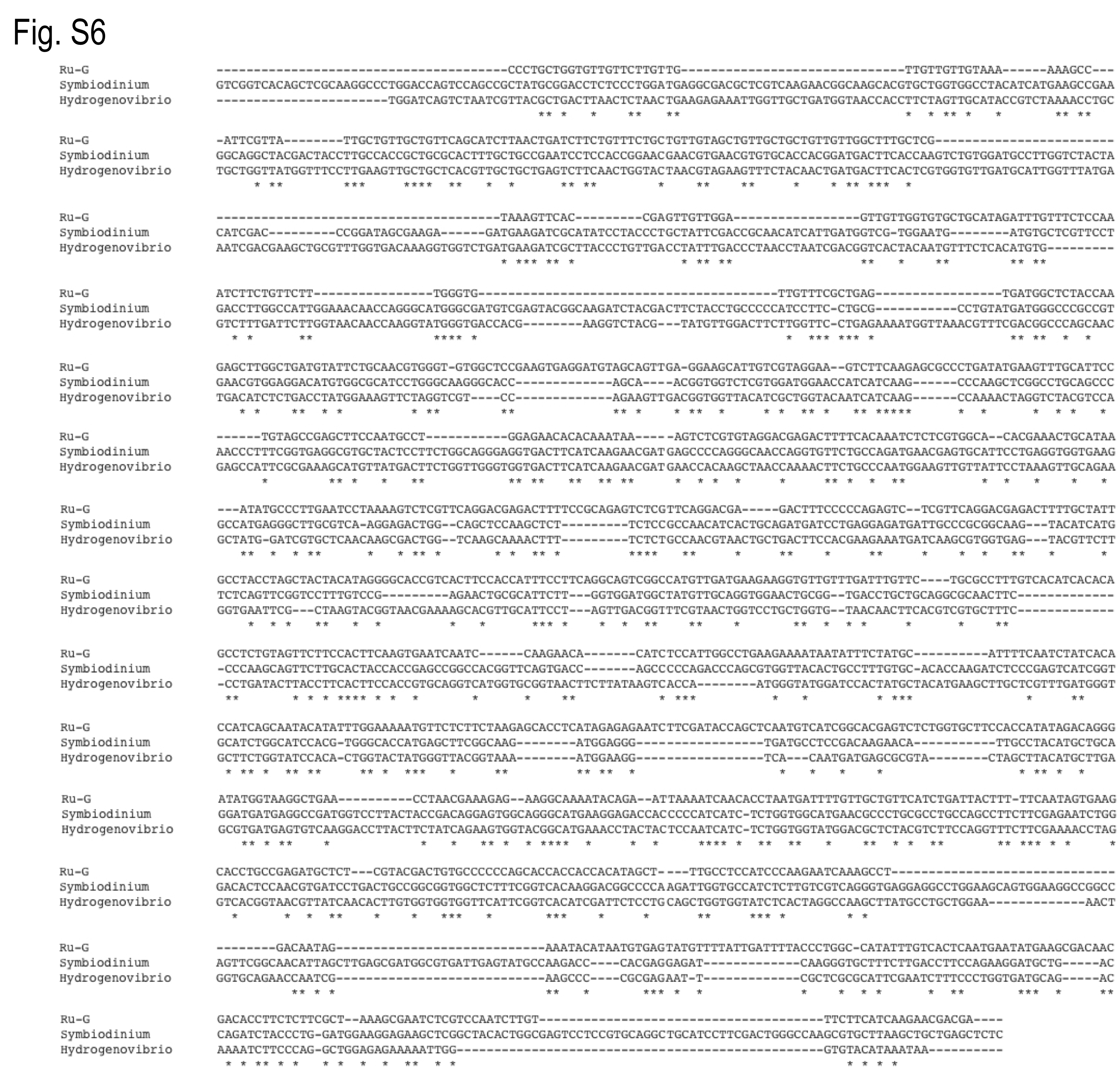

Supplement: Supplementary file 6 [file Image_6.JPEG]

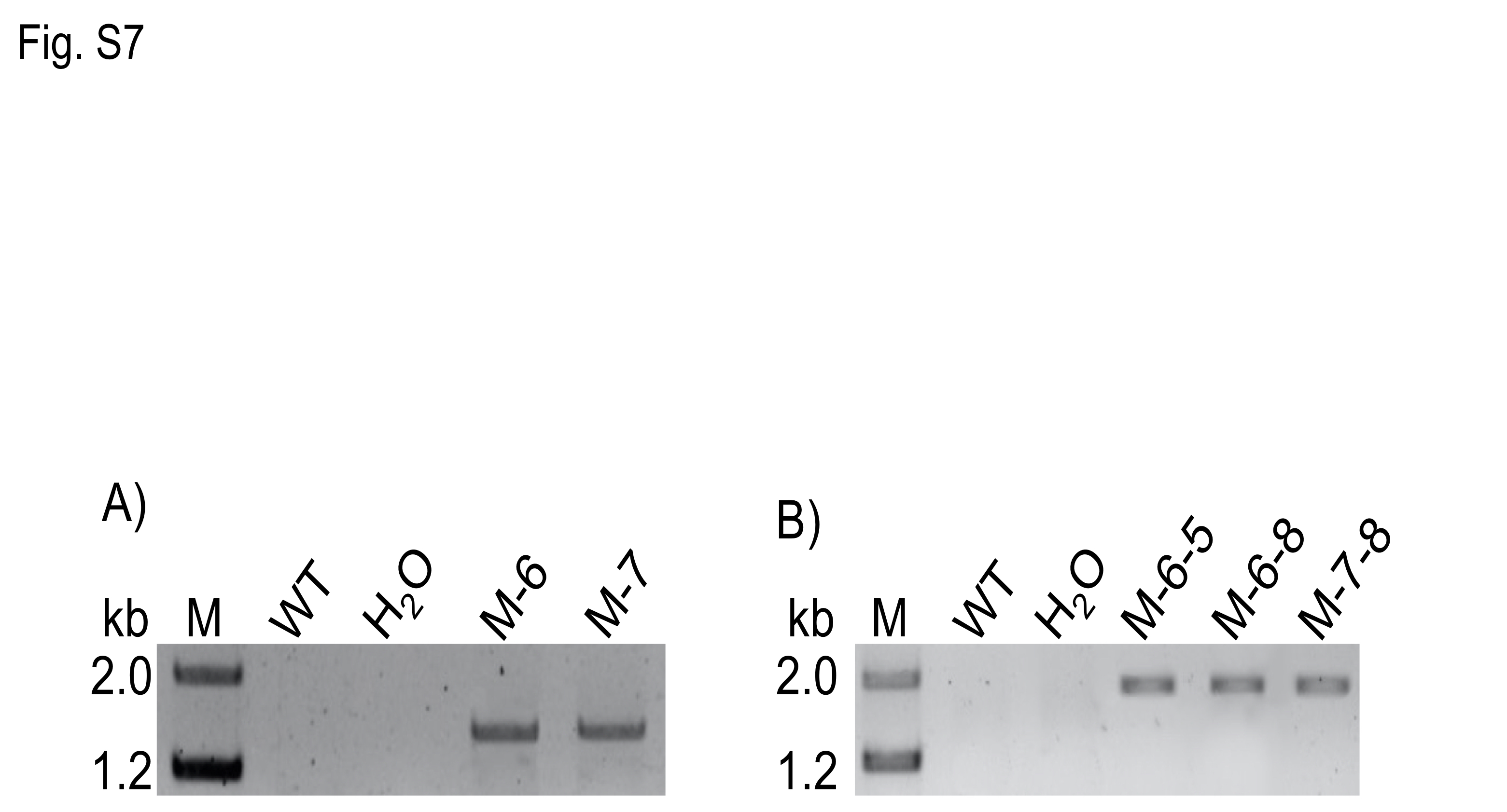

Supplement: Supplementary file 7 [file Image_7.JPEG]

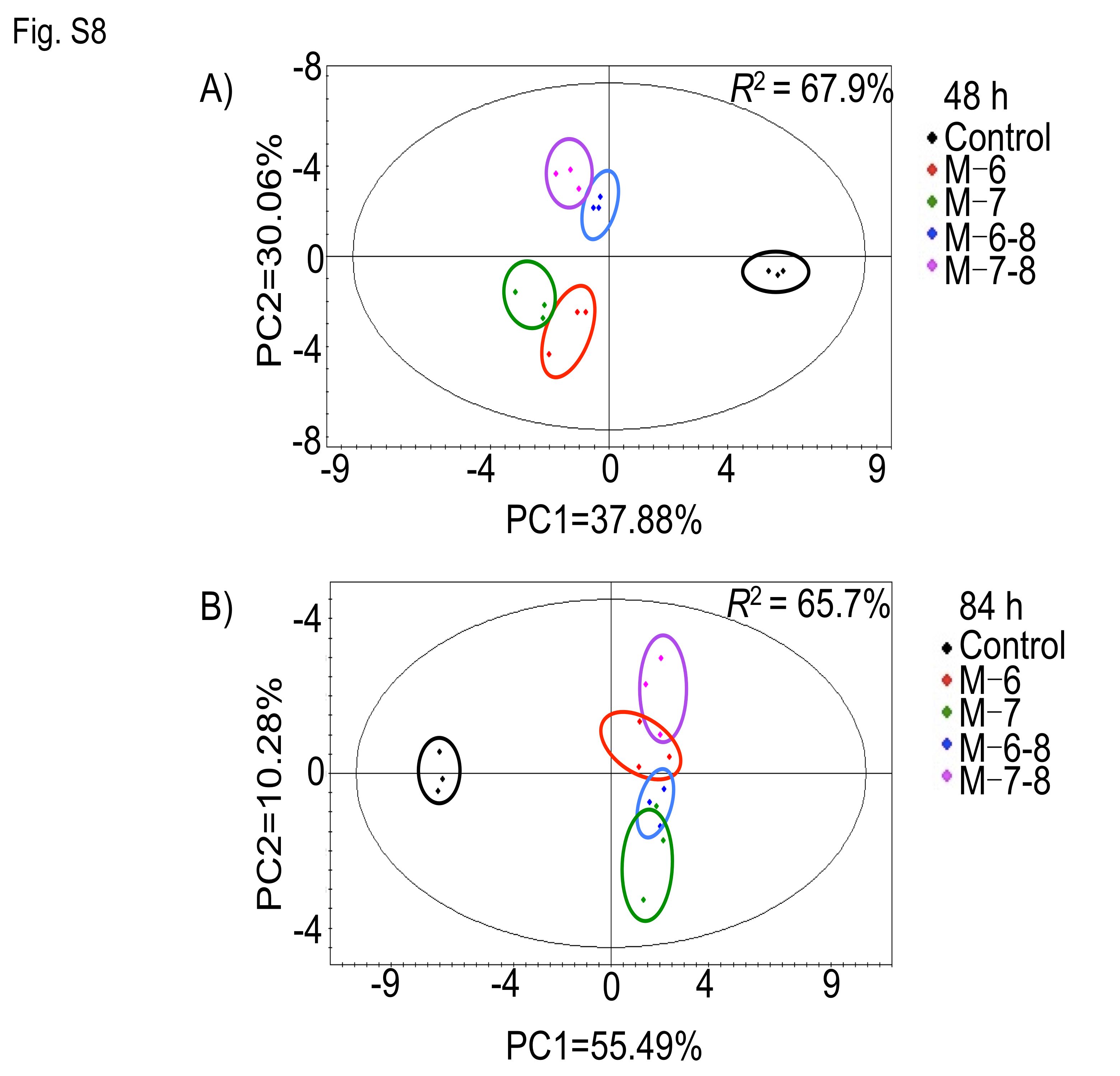

Supplement: Supplementary file 8 [file Image_8.JPEG]

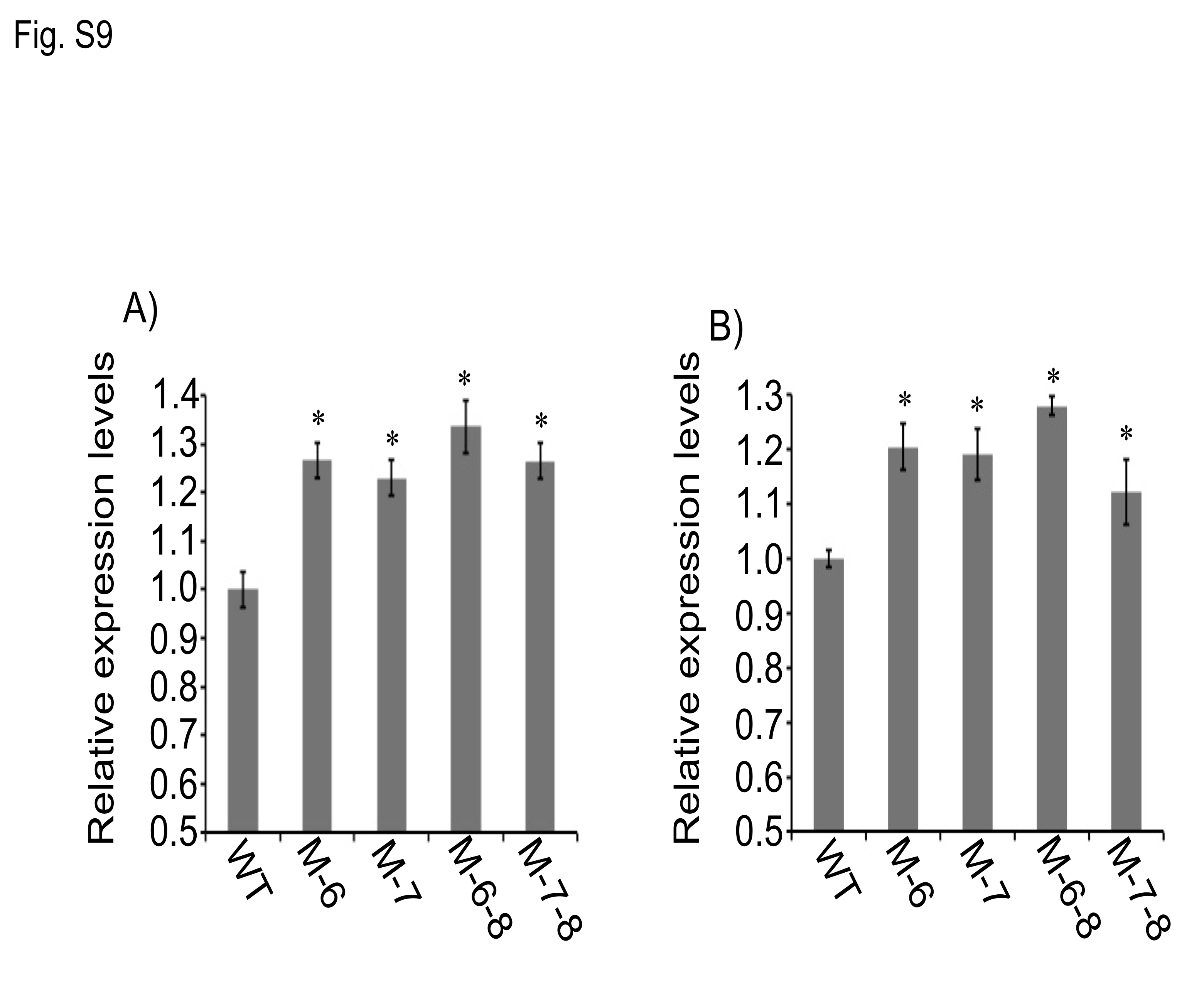

Supplement: Supplementary file 9 [file Image_9.JPEG]
