# Supplementary material for: Genetic Engineering of Crypthecodinium cohnii to Increase Growth and Lipid Accumulation
Source: Front Microbiol. 2018 Mar 19;9:492. doi: 10.3389/fmicb.2018.00492 (PMC5868476; doi:10.3389/fmicb.2018.00492)
Supplement: Supplementary file 10 [file Table_1.PDF]

Suppl. Table S1: Primers used in this study.

For C1 construction

Primer ID	Primer Sequences (5'-3')
18S-F1	TGTTTCAACACCCAACTTAT
18S-R	GTTAAGACTA GGACGGTATC
18S-R1	AGACGAGAGTGTCGTGCTCCACCAT GTTAAGACTAGGACGGTATC
Hyg-F2	ATGGT GGAGCACGAC ACT
Hyg-R2	CCGAATTAAT TCGGGGGA
18S-F3	ATCCAGATCCCCCGAATTAATTCGG CATAAACCATGCCAACTAGA
18S-R3	GAATTATTCA CCGAAGCAC

For rubisco cloning and mutant construction

Primer ID	Primer Sequences (5'-3')
Ru-F	CCCTGCTGGTGTTGTTCT
Ru-R	TCGTCGTTCTTGATGAAG
Ru-C-F1	CCCTGCTGGTGTTGTTCT
Ru--C-R1	AGACGAGAGTGTCGTGCTCCACCATCAAGTAGTCGTACCCGGACT
Hyg-F2	ATGGT GGAGCACGAC ACT
Hyg-R2	CCGAATTAAT TCGGGGGA
Ru-C-F3	ATCCAGATCCCCCGAATTAATTCGGCACTGACGGACGTGCCAT
Ru--C-R3	TCGTCGTTCTTGATGAAG
Ru-G-F1	CCCTGCTGGTGTTGTTCT
Ru-G-R1	AGACGAGAGTGTCGTGCTCCACCATCTCGAAAAACACAGCA
Ble-F2	ATGGT GGAGCACGAC ACT
Ble-R2	CCGAATTAAT TCGGGGGA
Ru-G-F3	ATCCAGATCCCCCGAATTAATTCGGATTTTGGATTTTCCCAGGGT
Ru-G-R3	TCGTCGTTCTTGATGAAG

For mutant analysis

Primer ID	Primer Sequences (5'-3')
Hyg-F3	ATGGCGTGATTTCATATGCGCGATTG
Ru-R1	TCGTCGTTCTTGATGAAG
Ble-F3	GCCAGAAGGAGCGCAGCCAAACCAG
P-F	CACCTACAAATGCCATCATTGC
Ru-R2	TTGTTTGCTTGGCGTTGTAAGCAGTAT GC

For RT-PCR

Primer ID	Primer Sequences (5'-3')
Ru-C-F	CATGAAGCCCAAGTCCGGGT
Ru-C-R	ATGTTGCGGTCGAACAGAGC
Ru-G-F	AGTCTTGTCCAGGAGGCCCAC
Ru-G-R	ACTGGCAAAAGGTCCCAGGAAT
ATP Syntha:	CAATCGGGAGTGCAAGTAGA
ATP Syntha:	ACGATGATCTGTCCAAGCAG
Ribosome-F	GTACATCATGGCTGTGCGA
Ribosome-R	CCCTTCAACACCTTCTTCCT
18S-F	AGTGGACTGCGGCTTAATTT
18S-R	CACCACCACCCATAGAATCA
