# Supplementary material for: Genetic Engineering of Crypthecodinium cohnii to Increase Growth and Lipid Accumulation
Source: Front Microbiol. 2018 Mar 19;9:492. doi: 10.3389/fmicb.2018.00492 (PMC5868476; doi:10.3389/fmicb.2018.00492)
Supplement: Supplementary file 11 [file Table_2.PDF]

Suppl. Table S2: LC-MS metabolomic dataset of C. cohnii*.

m/z	48h WT-1	48h WT-2	48h WT-3	48h M-6-1	48h M-6-2	48h M-6-3	48h M-7-1	48h M-7-2	48h M-7-3
AcCOA	0.11825053	0.12559349	0.17534653	0.01357009	0.05113067	0.07317071	0.1127561	0.01198752	0.21424085
NADPH	-0.8962387	-0.7224362	-0.5853351	-0.3246501	-0.5397829	-0.5515281	-0.1443742	-0.2069782	-0.3564725
NADP	0.32871053	0.53790884	0.54955598	-0.1050772	-0.1202443	-0.192475	0.03389177	-0.1976824	-0.0459546
NADH	-0.2503616	-0.1529712	-0.1206091	0.20066571	0.03465576	0.03379954	0.26356828	0.26106404	0.16360362
NAD	0.06748242	0.2337557	0.22708986	-0.0443257	-0.008246	-0.0052123	-0.0031081	-0.0081488	-0.0100275
ADP-Glucose	0.19195131	0.11334147	0.33738175	0.57382194	0.32746628	0.44217794	0.04508789	0.23773297	0.49489081
UDP-Glucose	0.18979276	0.48451706	0.41273459	-0.4540018	-0.2946334	-0.3111319	-0.2676597	-0.2375037	-0.0934041
ATP	-0.6713343	-0.3655332	-0.3927929	0.25480354	0.48107021	0.35370667	0.24545737	0.30045042	0.42115592
ADP	-0.6092211	-0.6594512	-0.5594911	0.36870676	0.52108891	0.54985764	0.56924381	0.45003887	0.41084393
COA	-0.623192	-0.7013744	-0.7876626	1.55587932	1.49862345	1.04716818	0.31394918	0.12442082	0.43236967
AMP	-0.2532098	-0.034334	0.03049227	0.30054404	0.30705001	0.31843432	1.09250825	0.93087654	1.08745389
FBP	0.46212367	0.93577088	0.95623498	-0.080246	-0.4435478	-0.4141852	-0.4956826	-0.8165225	-0.6167343
RiBP	0.18650325	0.31855333	0.21703265	-0.3786378	-0.3227846	-0.2709695	-0.2266347	-0.1598657	-0.6290722
G6P	0.0080005	-0.0056678	-0.007821	0.92516705	0.14834436	0.18267042	0.1712634	0.34218124	0.29107441
F6P	0.18781323	0.17864189	0.10897795	-0.0271441	-0.1916843	-0.0385618	-0.3173934	-0.309777	-0.3034101
R5P	-0.4033749	-0.6241932	-0.3783925	-0.4096887	-0.0294745	-0.1288365	-0.6192837	-0.6560227	-0.5518372
3PG	-0.2910971	-0.0852981	-0.1529174	0.20912804	0.01997698	0.09320437	0.22654188	0.28702904	0.3567074
DHAP	0.13433469	0.15961099	0.18012191	0.04448989	0.08035598	0.05473999	-0.3999914	-0.5144774	-0.200756
GAP	0.72805379	0.18213244	0.29276115	0.03437511	0.0722219	0.02962077	-0.1062941	-0.1238667	-0.1978673
PEP	-0.0406444	-0.2036348	-0.1563865	-0.3495235	-0.3975511	-0.3914595	-0.1291007	0.11589543	0.27107888
GLU	0.89213845	1.00434107	0.7234776	-0.4266211	-0.2487598	-0.2678196	-0.0963407	-1.2357336	-0.2873204
AKG	0.29291503	0.12535581	0.15340092	-0.2285805	-0.0912175	0.00253975	-0.0919607	-0.0570069	-0.0330181
OXA	-0.13877	0.02775945	0.05418161	-0.2304511	-0.1938739	-0.0376363	-0.1295027	0.1989253	-0.0486192
FUM	-0.2094269	-0.1174712	0.03332094	-0.8307006	-0.196968	-0.2746239	-0.0381952	-0.2034087	-0.0242407

m/z	48h M-6-8-1	48h M-6-8-2	48h M-6-8-3	48h M-7-8-1	48h M-7-8-2	48h M-7-8-3
AcCOA	-0.235317234	-0.24452301	-0.10465446	-0.044041	0.028656624	0.025318754
NADPH	-0.153792298	-0.12864987	0.185830521	-0.0683494	-0.09245392	0.062232258
NADP	-0.06824831	-0.23124201	-0.34509242	-0.2541351	-0.46489252	-0.31559589
NADH	0.023015701	-0.0075957	0.039339838	0.00323359	0.058502501	-0.05049974
NAD	-0.047571707	-0.16635472	-0.06265611	-0.201935	-0.39715969	-0.271739
ADP-Glucose	0.101753499	-0.39301119	-0.66261825	-0.3605897	-0.58565627	-0.13819729
UDP-Glucose	-0.118357039	-0.12336426	-0.09423606	-0.2482735	-0.27306946	-0.19539789
ATP	-0.102864778	-0.22393339	-0.00023202	-0.3468073	-0.52794633	-0.36640937
ADP	-0.278906137	-0.46357974	-0.46489583	-0.1905649	-0.28446833	-0.29311647
COA	-0.649334429	-0.48289442	-0.78584902	-0.6070903	-0.60869244	-0.60869244
AMP	-0.569947855	-0.73708225	-0.81658275	-0.4699837	-0.87704162	-0.91570362
FBP	-0.036937244	-0.24772451	-0.046367	-1.0436059	-1.08821219	-0.87422323
RiBP	-0.245743539	-0.15041595	-0.00532744	-0.1634951	-0.04160282	-0.35562116
G6P	-0.216754642	-0.02533924	-0.139848	-0.564812	-0.5395844	-0.44632126
F6P	-0.223959435	-0.60178409	-0.18080651	-0.4551107	-0.17470631	-0.07192771
R5P	-0.082707353	-0.59692026	-0.44491716	0.10836049	0.047440157	0.072570873
3PG	-0.015274883	-0.20291232	-0.02734298	-0.1526845	-0.0676658	-0.0680553
DHAP	0.129015037	-0.07767839	0.023116239	-0.2006036	-0.23203137	-0.03222023
GAP	0.009774199	-0.1488336	-0.21208113	-0.4644793	-0.03274766	-0.56363127
PEP	0.429723283	-0.48348863	0.036688922	0.15933052	-0.026415	0.132807076
GLU	-0.833722347	0.111429741	-0.56812743	0.05231093	0.046867995	-0.0558962
AKG	-0.208664111	-0.13657689	-0.00648286	-0.3334942	-0.2440508	-0.07442704
OXA	0.188085073	-0.04260519	0.12683005	0.14024128	0.151014971	0.004161034
FUM	0.015382282	-0.01136769	0.043011831	-0.0288537	0.039144674	-0.00057238

m/z	84h WT-1	48h WT-2	84h WT-3	84h M-6-1	84h M-6-2	84h M-6-3	84h M-7-1	84h M-7-2	84h M-7-3
AcCOA	-0.5786442	-0.6472208	-0.7471746	-0.0171254	0.03424395	0.06018031	0.11986234	0.12672416	0.16055209
NADPH	-0.7221348	-0.8417164	-0.8596383	0.28674865	0.27467581	0.23866098	0.32622823	0.42576391	0.47664508
NADP	-0.2470804	-0.3167124	-0.327021	0.02130719	0.19024235	0.00339325	0.29984269	0.31081381	0.04887194
NADH	0.02990149	0.29454221	0.15754128	0.06946482	0.10117883	0.07214979	-0.0438162	0.14644357	0.10117883
NAD	-0.0777101	-0.0475955	-0.0940154	-0.2484752	-0.3450381	-0.1709915	-0.2557613	-0.1340055	0.09963525
ADP-Glucose	-0.1609362	-0.0603879	-0.1609919	0.058822	-0.1609362	0.04238332	-0.353637	-0.5760294	-0.5760294
UDP-Glucose	-1.4318392	-1.3731509	-1.5908952	0.41577737	0.38744595	0.1006991	0.01487766	0.02363771	0.07041047
ATP	-0.4559098	-0.2615697	-0.3831057	0.26010061	0.24184483	0.16331555	0.05564597	0.09697349	-0.0352301
ADP	-0.838527	-0.7264542	-0.7717782	0.13037977	0.11242479	-0.0294509	0.08006237	0.29823549	0.25637605
COA	0.11184204	0.30519051	0.11184204	-0.6224244	-0.3194973	-0.087767	0.01840703	0.01229225	0.01184204
AMP	0.05510391	-0.0611538	-0.1165779	-0.1955433	-0.1555204	-0.0845415	-0.1173314	0.1085109	-0.0344101
FBP	1.43192928	0.93200288	0.34704038	-0.4119515	-0.6098909	-1.1737918	-0.9424662	-0.3714237	-0.7431574
RiBP	-1.0106472	-0.830075	-0.4581062	0.25238716	0.21709812	-0.1101829	0.45810622	0.59560975	-0.0195664
G6P	0.27180462	0.37308795	0.21533766	0.04503375	-0.0832903	-0.0428555	-0.0370673	0.06287024	0.02269259
F6P	-0.2272912	-0.2272912	-0.4496836	0.04968364	0.01271805	0.0522863	0.12729122	0.07528813	0.00175613
R5P	0.24594193	0.17997867	0.15041101	0.0923838	0.03384854	0.01967816	-0.3552308	-0.4049838	-0.3745135
3PG	0.23472802	0.25908722	0.24385551	-0.015854	-0.4353264	-0.2028169	-0.0399664	-0.2137464	-0.1116937
DHAP	-0.4624229	-0.8524262	-0.5505344	0.18064255	0.15240157	0.0122436	0.0641575	0.48232246	0.30928389
GAP	-0.1038706	-0.027055	-0.1345341	0.14405692	0.22086001	0.2180575	0.14934133	0.11873649	0.12494809
PEP	0.64742659	0.84159914	1.05018576	-0.2810202	0.08671163	-0.0453919	-0.3525734	-0.3772355	-0.5627911
GLU	-0.5416913	-0.4443322	-0.5159418	0.02196675	0.70961023	0.36981863	0.06011666	0.17357915	0.39313262
AKG	0.34880154	0.13012014	0.20557796	-0.0334517	-0.1436385	-0.1613285	-0.1193703	0.03860281	0.01563527
OXA	0.07523126	-0.0210766	-0.0018319	0.08112584	0.00113883	-0.0353544	0.12834098	0.02552062	-0.0967928
FUM	0.1502126	-0.2289077	-0.1109477	0.03656091	-0.1195314	-0.0169595	-0.0904794	-0.0998253	-0.0718176

m/z	84h M-6-8-1	84h M-6-8-2	84h M-6-8-3	84h M-7-8-1	84h M-7-8-2	84h M-7-8-3
AcCOA	0.20012563	0.283852282	0.106040003	0.315727957	0.119862336	0.17386424
NADPH	-0.00962238	0.227824528	0.318279479	0.349815053	0.33413343	0.2696447
NADP	0.06351424	0.1045731	0.193209844	0.009407559	0.013493739	0.0706153
NADH	0.183864192	-0.03116093	0.095419565	0.284453389	0.324392135	0.04252462
NAD	-0.13513461	-0.10897203	-0.00882283	-0.12812176	-0.23958844	-0.1377928
ADP-Glucose	-0.35363696	-0.57602938	-0.10498647	-0.00808738	-0.23513716	0.16093622
UDP-Glucose	0.377226111	0.216186751	0.069150327	0.17463958	0.284962995	0.2715201
ATP	0.235106228	0.118461309	0.276526155	0.35300546	-0.02512955	0.10443741
ADP	0.253891504	0.317489126	0.259289275	0.178451924	0.135112383	0.06702339
COA	-0.27642855	-0.3752067	-0.16421904	-0.19396639	-0.03297451	0.06237337
AMP	0.011650049	-0.04242662	-0.03956697	0.08881791	0.169159527	0.16612276
FBP	-0.48573579	-1.05349755	-1.17379178	-0.23792212	-0.20550064	-0.1427649
RiBP	0.367864379	0.211745177	0.574315257	0.036658471	0.08246216	0.40439026
G6P	0.018365357	-0.08329034	-0.19088462	-1.11723768	-0.67666508	-0.4315526
F6P	0.052816698	0.213281371	0.135278859	0.287281952	0.135278859	0.11012538
R5P	-0.13984177	-0.05796476	0.093043029	0.084054246	-0.00905516	-0.2266466
3PG	-0.03726726	0.036328773	-0.21801929	0.015681622	-0.22721823	0.00786212
DHAP	0.256745533	0.269791772	0.14865244	0.537577099	0.427059532	0.48730389
GAP	0.19991015	0.262451616	0.121631428	0.095801747	0.070447596	0.2798337
PEP	-0.12669201	-0.00639777	-0.40232645	-0.08546934	-0.16912727	-0.3044791
GLU	0.17243563	0.171741993	0.408735292	0.184677189	0.346292448	0.14800422
AKG	-0.08358962	0.015793492	-0.06791167	-0.21555269	-0.08834343	-0.2715086
OXA	-0.06374968	-0.01220641	-0.01692087	0.046350636	0.068098853	0.02273898
FUM	-0.05783457	0.020073555	-0.05325118	-0.09717167	0.086545089	-0.1097575

*The area of each compound obtained by LC-MS was divided by the cell numbers for normalization, and then standardization was made as follows: 1) For different time points, the data were divided by average of its row. 2) Log2 of the data were taken.
